# Context-Dependent Estrogenic Actions of (+)-Pisatin Produced in Elicited Green or Snow Pea (*Pisum sativum*)

**DOI:** 10.1021/acs.jafc.4c06409

**Published:** 2024-12-12

**Authors:** Jorge A. Belgodere, Megan C. Benz, G. Wills Kpeli, Jack R. Elliott, Steven Elliott, Jack D. North, Isaac J. Ponder, Peng Ma, Sophie R. Dietrich, Thomas Cheng, Khoa Nguyen, Syreeta L. Tilghman, John A. McLachlan, Binghao Zou, Muralidharan Anbalagan, Brian Rowan, Mark Mondrinos, Thomas E. Wiese, Van T. Hoang, Bridgette M. Collins-Burow, Elizabeth C. Martin, Matthew E. Burow, Stephen M. Boué

**Affiliations:** 1 Tulane Department of Medicine, Section of Hematology & Medical Oncology, Tulane University Health Science Center, New Orleans, Louisiana 70112, United States; 2 Department of Biological and Agricultural Engineering, Louisiana State University and Agricultural Center, Baton Rouge, Louisiana 70803, United States; 3 Tulane Cancer Center, Tulane University, New Orleans, Louisiana 70112, United States; 4 Department of Biomedical Engineering, Tulane University, New Orleans, Louisiana 70112, United States; 5 Xavier University School of Pharmacy, Xavier University, New Orleans, Louisiana 70125, United States; 6 Pharmaceutical Sciences Division, College of Pharmacy and Pharmaceutical Sciences, Florida A&M University, Tallahassee, Florida 32307, United States; 7 Department of Structural and Cellular Biology, Tulane University School of Medicine, New Orleans, Louisiana 70112, United States; 8 U.S. Department of Agriculture, Agricultural Research Service, Southern Regional Research Center, New Orleans, Louisiana 70124, United States

**Keywords:** legume, snow pea, green pea, phytoalexin, estrogenic, antiestrogenic, (+)-pisatin

## Abstract

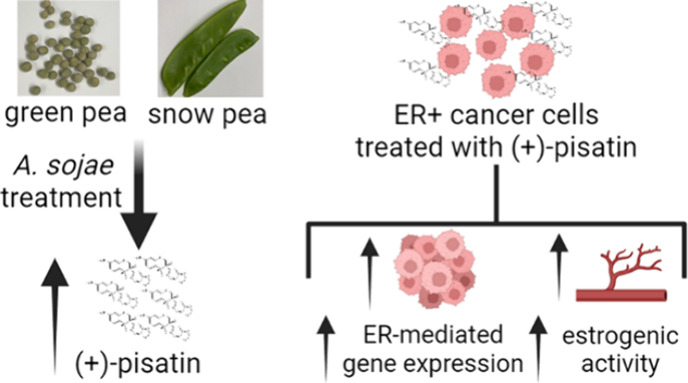

Legumes are a predominant source of isoflavones, termed phytoestrogens, that mimic 17β-estradiol (E2). Phytoalexins are inducible isoflavones produced in plants subjected to environmental stressors (e.g., UV, heat, or fungi). This study investigated estrogenic activity of snow and green peas elicited with *Aspergillus sojae*. Elicited extracts increased estrogenic activity and proliferation of breast cancer cells (MCF-7 or T47D) in a dose-dependent manner but exhibited antiestrogenic activity when combined with synthetic E2. HPLC analysis of elicited pea extracts identified (+)-pisatin as the primary phytoalexin, which was produced significantly (*p* < 0.0001) more in snow pea compared to green pea. RNA sequencing results suggested potential functional effects on endothelial cells and tissue vascularization. Indeed, (+)-pisatin enhanced metrics of network assembly and maturation in a microphysiological model of bulk tissue vasculogenesis. Thus, context-dependent functional effects of (+)-pisatin and pharmacologically similar phytoestrogens on the entire tissue microenvironment should be considered in preclinical investigation as potential therapeutic agents.

## 1 Introduction

Phytoestrogens are compounds produced by plants that possess weak estrogenic activity.^1^ They can be found in a wide variety of plants, including fruits and vegetables, but are most abundant in leguminous plants.^1^ Legumes are consumed in almost every diet throughout the world, and in addition to the seeds, many other parts of the plant are also edible, including the pods of some varieties. Soybeans have been demonstrated to contain high concentrations of the isoflavones daidzein and genistein,^2,3^ which are responsible for many of the health benefits of soy.^2−4^ Besides isoflavonoids, flavonoids also exert estrogenic activity, but usually at a much lower level of activity compared to that of isoflavonoids.^5^ Also, some flavonoids, including kaempferol and quercetin, can exhibit antiestrogenic activity,^6^ with several legumes being a source of these flavonoids.^7−9^ Coumestrol, a coumestan with high estrogenic activity in cell and animal assays, is also present in several legume seeds and sprouts.^6,10^

Several factors can alter legume isoflavone concentrations, including environmental conditions and germination.^11,12^ Changes in isoflavone concentrations also occur in response to stressors such as wounding, freezing, ultraviolet-light exposure, and exposure to microorganisms.^13−15^ In legumes, isoflavone phytoalexins are produced as plant defensive compounds.^15,16^ Careful examination of over 500 legumes has revealed that compounds belonging to six isoflavonoid classes, including isoflavones, isoflavonones, and coumestans, accumulate in tissues inoculated with fungi or microorganisms.^17^ In response to fungal stress, soybean produces the phytoalexins glyceollins I, II, and III that are derived from the isoflavone daidzein.^13−16^ These underutilized plant compounds could hold previously unknown potential for antioxidant activity, anti-inflammation activity, cholesterol-lowering ability, and even anticancer activity. Research in our laboratory has focused on the antiestrogenic activity of the soy glyceollins *in vitro*(18) and *in vivo*(19,20) with the glyceollins effective at inhibiting nonestrogen-dependent breast cancer cell growth. We have shown that *Aspergillus sojae* (*A. sojae*)-elicited red kidney beans produced the phytoalexins kievitone and phaseollin that displayed both estrogenic and antiestrogenic activities.^21^

Pea (*Pisum sativum* L.) is an annual, herbaceous, climbing plant belonging to the genus *Pisum* in the Leguminosae (Fabaceae) family, which can be classified into dry peas, green peas, and snow peas. Pea contains many different polyphenolic components, including phenolic acids, with antioxidant activity.^22−27^ Additionally, the four flavonoids identified in pea are daidzein, genistein, kaempferol, and apigenin.^25^ These flavonoids and other polyphenolics in pea have been shown to be both estrogenic and antiestrogenic;^5^ however, little information is available on the estrogenic activities of pea extracts and pea phytoalexins. Under conditions of stress, such as wounding, insect damage, or elicitor treatment, pea plants and seeds produce the phytoalexin pisatin.^28−33^ Pisatin can be produced in legumes (the (−) enantiomer) is present in some legumes, but the (+) enantiomer is primarily produced in peas (*Pisatum sativum* L.)) during abiotic^31^ or biotic elicitor treatment.^30,32,33^ Much research has been conducted on the biosynthesis of (+)-pisatin using (−) enantiomeric intermediates; however, only (+)-pisatin is produced in peas.^34^ Enantiomer differences can result in different bioactivity, such as antifungal activities,^35,36^ with (+) isomers exhibiting higher activity, compared to the (−) isomers.^37^ Pisatin has been shown to exhibit estrogenic activity by inducing MCF-7 cell proliferation,^38^ suggesting its ability to bind one or both estrogen receptors (ERα and ERβ) and potential to be antiestrogenic. Meanwhile, the positive enantiomer, (+)-pisatin, has shown low ERα binding affinity.^39^ The complexity of the (+)-pisatin bioactivity requires further investigation.

The estrogenic and antiestrogenic properties of phytoestrogens have led to the search for new plant sources and methods to induce phytoestrogens, particularly isoflavones, as both estrogen-targeted activities can be beneficial or harmful depending on the concentrations of isoflavones present and cell activity being examined. The goal of this work was to determine the effects of *Aspergillus sojae* treatment on estrogenic activity of the resultant extracts. Our specific objectives were as follows: (1) determine changes in chemical composition and identify phytoalexin compounds of green and snow peas after *A. sojae* elicitation, (2) evaluate estrogenic and proliferative activity of treated pea extracts in ER^+^ breast cancer cells, (3) determine the *A. sojae* effect on ER-mediated biological processes (PCR, Jess, docking and binding affinity, and RNA sequencing) in ER^+^ breast cancer cells, and (4) evaluate potential downstream effects (vasculogenesis).

## 2 Materials and Methods

### 2.1 Materials

Green pea seeds and snow pea (*Pisum sativum* L. var. macrocarpon Ser.) pods containing immature seeds were obtained from a local market (Goya brand, New Orleans, LA). HPLC-grade methanol, acetonitrile, ethanol, and water were used as solvents for the study and were purchased from Fisher Scientific (Pittsburgh, PA). A (+)-pisatin standard was obtained from Biosynth, and 17β-estradiol (E2) and dimethyl sulfoxide (DMSO) were obtained from Sigma-Aldrich (St. Louis, MO).

### 2.2 Snow and Green Pea Extraction and Elicitation

Snow pea pods with seeds and green pea seeds were surface-sterilized for 3 min in 70% ethanol, rinsed with deionized H_2_O (DI), 2 × 2 min DI rinses, and then soaked in sterile DI for 4 h prior to placement into Petri dishes (Corning, Durham, NC). Each Petri dish (100 × 15 mm) was lined with an autoclaved Whatman filter paper (Sigma-Aldrich, St. Louis, MO) moistened with 0.5 mL of distilled H_2_O. *Aspergillus sojae* (SRRC 1125) cultures were grown at 25 °C in the dark on potato dextrose agar (Sigma, St. Louis, MO). After 5 days, the inoculum was prepared by harvesting conidia (3.4 × 10^7^/mL) in 15 mL of sterile, distilled H_2_O. Green and snow pea pods with seeds were cut with a sterile razor, and then, 10 mL of the *A. sojae* spore suspension was applied to each cut surface for elicitor treatments. All samples were stored at 25 °C, in the dark, for 3 days and then transferred to −70 °C. Controls were whole (uncut) dry pods and seeds. After 3 days, elicited green pea seeds and snow pea pods with seeds were lyophilized and ground by using a Tekmar A-10 mill (Staufen, Germany). The ground pod/seed material (1 g) was extracted three times with 5 mL of methanol for 1 h using ultrasonic treatment (Branson 1510, Danbury, CT). The extracted solvent was filtered through 0.45 μm Nylon filters (Thermo, Waltham, MA), and the filtrate was used for analytical ultraperformance liquid chromatography electrospray ionization tandem mass spectrometry (UPLC-ESI-MS). For analysis of estrogenic activity, separate extracts using the same lyophilized source material were prepared in a similar manner. The resulting extracts were centrifuged at 10,000*g* for 20 min (Eppendorf 5415C, Hamburg, Germany), decanted, filtered, concentrated using a Thermo Savant SpeedVac SPD2030 (Thermo, Waltham, MA), and dissolved in DMSO at a concentration of 100 mg/mL.

### 2.3 Cell Culture

MCF-7 and T47D breast cancer cells were cultured in 150 cm^2^ culture flasks in Dulbecco’s modified Eagle’s medium (DMEM; Invitrogen, Carlsbad, CA) supplemented with 10% fetal bovine serum (FBS, Life Technologies, Inc., Gaithersburg, MD), basic minimum MEM essential (50×, Invitrogen, Carlsbad, CA) and MEM nonessential amino acids (100×, Invitrogen, Carlsbad, CA), sodium pyruvate (100×, Invitrogen, Carlsbad, CA), antimycotic–antibiotic (10,000 U/mL penicillin G sodium, 10,000 μg/mL streptomycin sulfate, and 25 μg/mL amphotericin B as Fungizone, Invitrogen, Carlsbad, CA), and human recombinant insulin (4 mg/mL, Invitrogen, Carlsbad, CA). The culture flasks were maintained in 5% CO_2_ at 37 °C.

### 2.4 ERE-Luciferase Assay

Pea extracts (nonelicited or *A. sojae*-elicited) or isolated (+)-pisatin was prepared in DMSO at a concentration of 10 mM (stock solution). The final concentration of DMSO was adjusted to less than 0.1% (v/v). Both MCF-7 and T47D cells were placed in stripped media: phenol red-free DMEM (Gibco, Billings, MT) supplemented with 5% dextran-coated charcoal-treated (DCC, HyClone, Logan, UT) FBS for 48 h prior to plating. Cells were plated in 24-well plates (Corning, Corning, NY) at 5 × 10^5^ cells/well and allowed to attach overnight. The next day, cells were transfected with the ER(2)-luc plasmid (Panomics, Fremont, CA) using an Effectene transfection reagent (Qiagen, Hilden, Germany), following the manufacturer’s protocol. After 6 h, cells were treated and incubated at 37 °C with various concentrations of nonelicited or *A. sojae*-elicited pea extracts or isolated (+)-pisatin. DMSO, E2 (0.1 nM), or fulvestrant (ICI 182, 780; 1 μM; Sigma, St. Louis, MO) were used as controls. Combinations of extracts and ICI or E2 were also evaluated. Media were removed after 18 h, replaced with 200 μL/well of 1× lysis buffer (Promega, Madison, WI), incubated for 15 min at room temperature, and then pelleted by centrifugation at 15,000*g* for 5 min. Cell extracts were normalized for protein concentration using a Bio-Rad reagent following the supplied protocol (Bio-Rad Laboratories, Hercules, CA). The luciferase activity was determined using a 1× luciferase assay substrate (Promega, Madison, WI) in a Lumat LB 9510 luminometer (Berthold, Bad Wildbad, Germany). Reported data are the mean ± SEM of three independent experiments.

### 2.5 Cell Proliferation Assay

Cell proliferation was based on previously published methods.^40−42^ Briefly, cells were placed in phenol red-free DMEM supplemented with 5% DCC FBS for 7 days prior to plating. The cells were plated in 96-well plates (Corning, Corning, NY) at a density of 4.5 × 10^3^ cells/well. After 24 h, cells were dosed with 100 μL/well of media consisting of various concentrations of nonelicited or *A. sojae*-elicited pea extracts or isolated (+)-pisatin. DMSO, E2 (0.1 nM), and ICI (100 μM) were controls. Combinations of extracts and ICI or E2 were also evaluated. The cells were redosed on day 4 and cell proliferation was measured on day 7, when positive controls reached 90–100% confluence. An Alamar Blue dye (Thermo Fisher Scientific, Waltham, MA) was added to the medium (10 μL/well), and the plates were incubated for 3 h at 37 °C with 5% CO_2_. Fluorescence was evaluated at 560 nm excitation and 595 nm emission by using an HTS7000 series bioassay reader (PerkinElmer, Boston, MA). Reported data are the mean ± SEM of three independent experiments.

### 2.6 Quantitative PCR and PCR Array

MCF-7 or T47D cells were seeded into 25 cm^2^ culture flasks (VWR, Radnor, PA) at ∼5000 cells/cm^2^ and grown until 70–80% confluency. Once confluent, media were aspirated, cells were washed with PBS three times, 5 mL of stripped media (phenol red-free DMEM, 5% DCC FBS, penicillin streptomycin, essential amino acids, nonessential amino acids, sodium pyruvate, and Glutamax) was added, and cells were incubated for 48 h. After 48 h, 5 μL of DMSO, E2 (100 nM), pea extracts, (+)-pisatin (10 mM), or ICI was added to the corresponding flasks and incubated for 4 h. After 4 h, 5 μL of DMSO was added to E2, DMSO, and (+)-pisatin flasks, and 5 μL of E2 or (+)-pisatin was added to the ICI flasks. After 24 h, cell pellets were collected, and total RNA was extracted using the RNeasy mini kit (Qiagen, Hilden, Germany) and reverse-transcribed to complementary DNA (cDNA) using an iScript cDNA supermix (Bio-Rad, Hercules, CA), as per the manufacturer's instructions. Quantitative PCR was performed using the Bio-Rad CFX Connect real-time system (v4.006; Bio-Rad, Hercules, CA) and a Bio-Rad IQ SYBR green supermix (Bio-Rad, Hercules, CA) as per the manufacturer’s protocol. Expression was calculated using the ΔΔ(Ct) method and reference gene RPL13a. Primer sequences are presented in Table S1. Samples were normalized to vehicle control gene expression (DMSO) and designated as 1. Biological triplicate experiments were run for each sample. For the PCR array, RNA was reverse-transcribed to first-strand cDNA using the RT2 first-strand kit (Qiagen, Hilden, Germany), mixed with the RT2 SYBR Green Mastermix (Qiagen, Hilden, Germany), and then aliquoted into the wells of the human cancer PathwayFinder RT^2^ Profiler PCR array (Qiagen, Hilden, Germany), following the manufacturer's instructions. qRT-PCR was performed, and expression was calculated using the ΔΔ(Ct) method and reference gene β-actin.

### 2.7 Isolation and Characterization of (+)-Pisatin from Elicited Snow and Green Peas

For semipreparative isolation of (+)-pisatin, 0.5 kg of green and snow pea pods and seeds was prepared as described above and extracted using 2 L of methanol under rotary evaporation for 2 h (between 35 and 45 °C) to concentrate the methanolic extracts before semipreparative HPLC. (+)-Pisatin was isolated using techniques developed at the Southern Regional Research Center (ARS, USDA, New Orleans, LA).^23^ For the isolation of the phytoalexins, semipreparative HPLC-UV (UV detection) was utilized. The column was a Whatman ODS-2 10 mm × 500 mm using a flow rate of 3.0 mL/min with the following solvent system: A = acetonitrile, B = water; 5% A for 15 min and then 5% A to 90% A in 40 min followed by holding at 90% A for 20 min. Acetonitrile was removed from fractions containing phytoalexins and lyophilized to remove water. Phytoalexin purities >98% were achieved based on HPLC-UV analyses.

Identification of (+)-pisatin in green pea and snow pea samples was achieved by comparing its retention time, UV–vis, MS, and high-energy (MS^E^) spectra with those of the pisatin standard. Analyses were performed on a UPLC system (Acquity H-Class, Waters Corp., Milford, MA) with a binary solvent manager, a column manager, and a sample manager. The positive ion electrospray mass spectra of (+)-pisatin from snow peas, green peas, and the standard are shown in Figure S1. The MS spectrum displays the predominant [M + H–H_2_O]^+^ ion at *m*/*z* 297.0857 and the lower abundant ion [M + H]^+^ at *m*/*z* 315.0854. The loss of water in ESI-MS spectra has been noted in other pterocarpans such as the glyceollins and occurs with the loss of the 6a-hydroxyl group.^43^ The ESI-MS/MS spectra for (+)-pisatin are shown in Figure S2. The predominant product ion at *m*/*z* 225.0551 is [M + H–C_2_H_2_O_4_]^+^ from the loss of water, the methoxy group, and the methylenedioxy ring. The sample was separated using a Waters Acquity BEH C-18 column (150 mm × 2.1 mm, 1.7 mm) with a column temperature of 40 °C. The mobile phase consisted of water containing 0.1% formic acid (A) and acetonitrile containing 0.1% formic acid (B) with a flow rate of 0.3 mL/min. The gradient elution program was as follows: mobile phase B from 10 to 30% (0–10.00 min), from 30 to 80% (10.00–15.00 min), from 80 to 10% (15.00–17.00 min), and maintained at 10% (17.00–20.00 min). The temperature of the column and autosampler was controlled at 40 and 20 °C, respectively, and the injection volume was 5 μL. A Xevo G2-XS QTOF mass spectrometer (Waters Corp., Milford, MA) equipped with an electrospray ionization (ESI) source was operated in positive ionization mode. The mass spectrometer parameters were as follows: capillary voltage, 2.00 kV; sampling cone, 15 V; extraction cone, 4.0 V; source temperature, 120 °C; desolvation temperature, 300 °C; cone gas flow rate, 50 L/h; desolvation gas flow rate, 600 L/h. Leucine-enkephalin was used as the lock mass, generating an [M + H]^+^ ion (*m*/*z* 556.2771) to ensure accuracy during MS analysis. The data were collected in centroid mode by MS^E^ acquisition. The MS^E^ experiment in two scan functions was carried out as follows: function 1 (low energy): mass-scan range, 100–600 Da; scan time, 0.2 s; interscan time, 0.05 s; collision energy, 2 eV; function 2 (high energy): mass-scan range, 100–600 Da; scan time, 0.2 s; interscan time, 0.05 s; collision energy ramp, 20–30 eV. MassLynx and UNIFI software (Waters Corp., Milford, MA) was used for the postacquisition analysis (Waters Corp., Milford, MA). The nuclear magnetic resonance (NMR) spectra were recorded on a Bruker DRX-500 system (Karlsruhe, Germany). ^1^H NMR (CDCl_3_) δ ppm: 3.74 (3H, s), 3.99 (1H, d, *J* = 11.5 Hz), 4.17 (1H, d, *J* = 11.5 Hz), 5.23 (1H, s), 5.89 (1H, d, *J* = 1.2 Hz), 5.92 (1H, d, *J* = 1.2 Hz), 6.38 (1H, s), 6.44 (1H, d, *J* = 2.4 Hz), 6.63 (1H, dd, *J* = 2.5 and 8.6 Hz), 6.79 (1H, s), 7.33 (1H, d, *J* = 8.5 Hz). ^13^C NMR (CDCl_3_) δ ppm: 55.6, 69.4, 76.0, 84.7, 93.8, 101.5, 101.6, 104.3, 109.4, 113.5, 120.9, 132.6, 141.9, 149.2, 154.2, 156.1, 160.8.

UPLC analyses were performed on a Waters Acquity H-Class instrument with a PDA detector. A Waters Acquity BEH C-18 column (150 mm × 2.1 mm, 1.7 mm) was used at a flow rate of 0.3 mL/min with a 5 mL injection volume. Peak areas of (+)-pisatin were quantified at 309 nm. A linear calibration curve (*R*^2^ > 0.99) for (+)-pisatin was prepared between a range of 4–200 μg/mL. Reported data are the mean ± SEM of three independent experiments. (+)-Pisatin concentrations were reported as μg/g of snow peas and green peas on a dry weight basis.

### 2.8 Capillary Western Blot (Jess) Analysis

MCF-7 cells were seeded into 6-well plates (Corning, Corning, NY) at ∼26,000 cells/cm^2^ and grown until 70–80% confluency. Once confluent, the medium was aspirated, cells were washed with PBS three times, 2 mL of the stripped medium was added, and cells were incubated for 48 h. After 48 h, treatments of DMSO (2 μL) and (+)-pisatin (10 μM) were added to the corresponding wells and incubated at consecutive time points of 0, 1, 6, and 24 h. After each time point, media were aspirated, and cells were washed with 1 mL of cold PBS, aspirated, and then scraped using a cell scraper (VWR, Radnor, PA). The mammalian protein extraction reagent lysis buffer (MPER buffer, Thermo Fisher Scientific, Waltham, MA) was made by adding 50 μL of protease and phosphatase inhibitor cocktail (100×, Thermo Fisher Scientific, Waltham, MA) to 5 mL of MPER. A 100 μL amount of the MPER buffer was added to each well, agitated to ensure lysing, and then transferred to 1.5 mL Eppendorf tubes (Sigma-Aldrich, St. Louis, MO). Tubes were then briefly vortexed, incubated on ice for 8 min, and centrifuged at 14,000 rpm for 10 min at 4 °C. The supernatant was transferred to a new tube and diluted to a final concentration of 3 μg/μL.

Capillary Western blot analyses were performed using the ProteinSimple Jess system using the Wes/Jess separation module (12–230 kDa) (ProteinSimple, San Jose, CA) that contains capillary cartridges and prefilled microplates with a split running buffer, wash buffer, and sample buffer. The module also has EZ Standard Pack 1 with a 5× fluorescent master mix, biotinylated ladder, and DTT. The antirabbit detection master kit includes an antirabbit secondary antibody, an antibody diluent, streptavidin-HRP, luminol-S, peroxide, and a wash buffer. All were purchased from ProteinSimple and used following the manufacturer's protocol. Briefly, a 0.1× sample buffer was used to dilute MCF-7 lysates to 1.25 μg/μL. Then, 1 part of the 5× fluorescent master mix (which included the 5× fluorescent standard, 5× sample buffer, and 200 mM DTT) was mixed with 4 parts of the diluted samples and heated for 5 min at 95 °C to denature. Since the molecular weight ladder is only present on the first capillary and each capillary is independent, the system control proteins in the fluorescent master mix function as a “ruler” to standardize the distance for each capillary.^44,45^ Following the denaturation process, the primary antibodies (ERα 1:500 dilution), secondary antibodies conjugated with HRP, blocking reagent, and chemiluminescent substrate were added to the appropriate wells in 12–230 kDa separation plates. Antibodies used were specific for ERα (Millipore Sigma, Burlington, MA) and pERα (Cell Signaling, Danvers, MA). For every experiment, molecular weight standards were supplied via a biotinylated ladder. After loading the assay plate, the fully automated capillary system performed separation electrophoresis and immunodetection. A CCD camera in the Jess collects the data. The Compass for Simple Western program (ProteinSimple, San Jose, CA) was used to analyze the data. Areas under the peaks in Jess were noted to present the relative amount of protein. Reported data are the mean ± SEM of three independent experiments.

### 2.9 Docking Models of (+)-Pisatin to ERα

(+)-Pisatin has two chiral centers within the conjugated, multiring structure resulting in four possible enantiomers. Of these configurations, only the RR configuration is found in the plants of the pea family; therefore, this enantiomer was used for ligand–receptor modeling (docking) studies. All molecular modeling in this study was done by using Molecular Operating Environment (MOE) 2020.8 software (Chemical Computing Group, Montreal, QC, Canada). The RR configuration or (+)-pisatin 3D structure was created and optimized in MOE. Docking of (+)-pisatin into the crystal structures of the human ERα ligand-binding domain in complex with E2 (PDB ID 1ERE) and in complex with 4OH-tamoxifen (PDB ID 3ERT) was performed in the Docking function of MOE using the Triangle Matcher placement method, London dG initial scoring, rigid receptor refinement, and GBVI/WSA dG final scoring saving the five most energetically favorable poses. These docking simulations used the A Chain of each crystal structure that was protonated using the Structure Preparation and Protinate 3D functions of MOE. Docking procedures were validated by replacing the E2 and 4OH-tamoxifen into the 1ERE and 3ERT ligand pockets in the same binding pose as the crystal structures (Figure S6). The “best scoring” (*S* score) poses of (+)-pisatin docked with 1ERE and 3ERT were retained for analysis and comparison. The ER agonist and antagonist binding pockets are illustrated by MOE as Connolly Channels, where green represents the hydrophobic regions and purple represents the polar regions. Key amino acids are shown in the binding pocket.

### 2.10 RNA Sequencing

Extracted RNA was quantified and assessed for integrity using a Bioanalyzer (Agilent Technologies, Santa Clara, CA). The total RNA was extracted using a Direct-zol RNA purification kit (Zymo Research, Irvine, CA). The mRNA was then purified from total RNA using oligo(dT)-attached magnetic beads and fragmented into small pieces. The fragmented mRNA was synthesized into first-strand cDNA using random primers. The second strand of cDNA was synthesized with dUTP instead of dTTP. The cDNA thus synthesized was subjected to end-repair and 3′ adenylation. Subsequently, adaptors were ligated to the ends of this 3′-adenylated cDNA fragment. A U-labeled second-strand template was digested with uracil-DNA-glycosylase (UDG), PCR amplification was performed, and sequencing on the DNBSEQ (DNBSEQ Technology) platform was performed. Unbiased pathway analysis was performed through Enrichr pathways and the MSigDB Hallmark gene set. Genes included in the analysis were all significantly changed genes with adjusted *p*-values of *p* < 0.05.^46−48^ The data discussed in this publication have been deposited in NCBI’s Gene Expression Omnibus^49^ and are accessible through GEO Series accession number GSE277758.

### 2.11 (+)-Pisatin-Mediated Vasculogenesis

#### 2.11.1 Cell Culture

Human umbilical vein endothelial cells (HUVEC, ATCC, Manassas, VA) were maintained in a vascular cell basal medium supplemented with endothelial cell growth kit-VEGF (ATCC, Manassas, VA) and 2% FBS (ATCC, Manassas, VA). Human lung fibroblasts (HLF, ATCC, Manassas, VA) were maintained in a fibroblast basal medium supplemented with a fibroblast growth lit-low serum (ATCC, Manassas, VA) and 2% FBS. All culture media contained 1% antibiotic–antimycotic (Corning, Corning, NY). Cells were maintained in a humidified tissue culture incubator maintained at 37 °C and 5% CO_2_. Cells were harvested at 80–90% confluency, and experiments were performed using cells between passages 3 and 6.

#### 2.11.2 Ki67 Immunofluorescence Staining and Quantification

HUVECs were seeded at 10,000 cells per well in 48-well tissue culture plates and cultured for 24 h. The tissues are treated with (+)-pisatin at various concentrations in an endothelial cell growth medium for an additional 24 h. 2D plated HUVECs were fixed in 4% paraformaldehyde (PFA, Polysciences) for 15–20 min and washed with PBS. The samples were blocked and permeabilized with 1% bovine serum albumin (Sigma-Aldrich, St. Louis, MO) and 0.1% Triton X-100 (Sigma) for 15 min and incubated with primary antibodies against the Ki76 index marker (Abcam, Cambridge, United Kingdom) for 2 h at room temperature at a 1:100 concentration in 0.1% BSA in PBS. After washing, the secondary antibody goat antirabbit IgG (Alexa Fluor 594, Abcam, Cambridge, United Kingdom) was dissolved in 0.1% BSA solution at a dilution of 1:500 along with DAPI (Hoechst 33342, Thermo Fisher, Scientific, Waltham, MA) and phalloidin (Alexa488, Invitrogen, Thermo Fisher Scientific) at 1:500 dilutions at room temperature covered from the dark. Stained tissues were washed with PBS and stored at 4 °C and protected from light until imaging. To quantify the Ki67 index, cell nuclei of spheroids (stained with Hoechst 33342) were segmented into discrete regions of interest (ROI) using a watershed transformation.^50^ Mean fluorescence within each nucleus-ROI was computed, and a minimum threshold was set to classify cells as positive or negative for Ki67. The Ki67 index was reported as the fraction of positively fluorescing cells in each image. The cell density was quantified using DAPI-positive nuclei. All image analysis was completed in MATLAB (R2023b).

#### 2.11.3 Stereolithography Printing, Postprocessing, and PDMS Casting

Device molds were drafted as 3D drawings using SolidWorks (SolidWorks Corp., Waltham, MA) or Fusion 360 (Autodesk, Inc., San Francisco, CA). Molds were created in a top-down view and printed using a Form 3B SLA printer (Formlabs, Inc., Somerville, MA). Final designs were exported as .STL files to Formlab’s PreForm software. Formlab’s PreForm software was used to prepare 3D drawing files for printing. The part file was oriented so that the mold base was parallel with the printer’s build platform. Print supports were autogenerated within PreForm with a 0.65 mm touchpoint size and a 1.30 support density. All molds were printed using Formlab’s proprietary “Clear” SLA resins.

Completed prints were washed in isopropyl alcohol (IPA, Fisher) according to Formlabs protocols for the resins used and then washed in IPA for 20 min in the Form Wash (Formlabs, Inc., Somerville, MA). Post wash, molds were dried until IPA was completely evaporated. Molds were then dried and cured under UV light (Form Cure, Formlabs, Inc., Somerville, MA) at 60 °C for 15 min. Warping was caused by distortion of the printing process, and the curing process was corrected by first baking molds at 130 °C for 2 h. For the last 30 min of baking time, two stainless-steel jeweler’s blocks were added to the oven to heat. Molds were removed and placed between two jeweler’s blocks with or without clamping. Flatness of the parts was assessed visually relative to a straight edge. We used a commercial painting airbrush (Badger Airbrush Co., Bellwood, IL) to coat molds with a lacquer thinner (Klean-Strip, Memphis, TN). Parts were dried for 15 min while preparing an automotive clear coat (Sherman Williams, Cleveland, OH) mixed at a 1:4 ratio of the hardener to clear coat. The mixture was airbrushed from approximately 8 in. away using a continuous back and forth motion and then a continuous up and down motion until a thin layer of clear coat was visible. Four layers were applied with the mold rotated 90° between applications. Coated molds were dried for 6 h before silanization according to standard protocols for soft lithography.^51^

Polydimethylsiloxane (PDMS) (Sylgard 184, Ellsworth Chemical, Germantown, WI) was mixed at a 1:10 ratio of the PDMS curing agent to PDMS elastomer by weight and degassed. PDMS soft lithography was carried out according to standard protocols used for silicon master molds.^52^ To produce PDMS molds with two flat surfaces, we applied a cleaned 2 × 3 in glass slide to the top of the mold to sandwich the uncured PDMS, carefully avoiding bubble formation. A jeweler’s block was placed on top of the slide to ensure a tight seal, and filled molds were baked at 60 °C for at least 4 h.

#### 2.11.4 Vasculogenesis Assay

Devices were exposed to UV light in a cell culture hood for 20 min. The surfaces of the chambers of the constrained device that house bulk 3D tissues were functionalized for extracellular matrix (ECM) hydrogel anchorage using a modified version of the polydopamine (PDA, Sigma-Aldrich, St. Louis, MO) coating method as previously described by Park et al.^53^ Briefly, a 5 mg/mL dopamine solution was prepared by mixing dopamine hydrochloride (Sigma-Aldrich, St. Louis, MO) with 10 mM Tris-hydrochloride (Sigma-Aldrich, St. Louis, MO). Dopamine solution was sterile filtered and pipetted into the inner well of the constrained device. Devices were incubated for 2 h at room temperature under ultraviolet irradiation, aspirated, and washed with ultrapure water. PDA-treated devices were used within 1 week of coating. Biological sex female HLF and HUVEC (2 × 10^6^ cells/mL each) were admixed in 2.2 mg/mL collagen I (Corning, Corning, NY), 5 mg/mL fibrinogen (Sigma-Aldrich, St. Louis, MO), and 1 U/mL thrombin (Sigma-Aldrich, St. Louis, MO). This cell-inoculated hydrogel precursor was injected into the inner well of the constrained device and incubated for 15 min. The outer chambers were filled with an endothelial cell growth medium (ATCC) supplemented with 25 μg/mL aprotinin (EMD Millipore, Burlington, MA) and 2% FBS and replenished after 2 days. After 3 days of culture, the tissues were washed twice with PBS and replaced with a phenol-free endothelial cell growth medium supplemented with a 2% charcoal-stripped serum (CSS, GIBCO) and 25 μg/mL aprotinin and cultured for additional 3 days. Experimental groups were treated with (+)-pisatin at a 10 μM concentration with or without ICI at a 1 μM concentration. ICI groups were treated at a 1 μM concentration. The medium was replenished every 2 days; bulk vasculogenesis tissues were cultured for a total of 6 days with the initial seeding described as day 0.

#### 2.11.5 Immunofluorescence Staining in Bulk Vasculogenesis Tissues

Bulk tissues in devices were stained using adaptations of a previously reported protocol.^54−56^ Briefly, tissues were fixed by loading 4% PFA in the outer chambers of the device and incubating them for 1 h at room temperature. Devices were washed with PBS and stored at 4 °C prior to staining. Tissues were stained using 4 μL/mL DAPI to label nuclei, 4 μL/mL phalloidins to label actin in all cells, and 10 μL/mL DyLight 594-conjugated Ulex europeaus agglutinin I (UEA-1, Vector Laboratories, Newark, CA) to specifically label microvascular networks.^57^ The staining cocktail was prepared in 1× PBS with 0.2% Triton X-100 and 1% BSA. The tissues were incubated with the staining cocktail solution on a rocker for 1 h at room temperature prior to washing with PBS. Stained tissues were loaded with PBS and stored at 4 °C protected from light until imaging.

#### 2.11.6 Imaging and Analysis

Stained tissues were fixed in a position on a glass slide and imaged on an inverted Nikon C2 laser scanning confocal microscope (LSCM) equipped with a Nikon DS-FI3 camera. A large composite containing a 2 × 2 and 3 × 3 stitched image was acquired using a 10× magnification objective lens from each sample of bulk vasculogenesis and 2D Ki67 index proliferation experiments, respectively. For bulk vasculogenesis tissues, five evenly spaced *z*-slices of the stitched images were acquired. Max intensity projections of *Z*-stacks from vasculogenesis samples were exported as TIFFs for image analysis. All vascular morphometric analysis was completed in MATLAB (vR2021b, MathWorks, Natick, MA). Vascular network images were smoothed using an edge preserving filter with a Gaussian kernel, and a threshold was applied to remove the remaining low-intensity noise. We used a pretrained deep neural network to denoise each image, and adaptive histogram equalization was used to standardize contrast across the image set. We then segmented preprocessed images and quantified morphometric parameters using an open-source automated segmentation tool.^58^

### 2.12 Statistical Analysis

Statistical analyses were performed using GraphPad Prism (version 10.0.2, GraphPad Software, San Diego, CA). One- or two-way ANOVA with multiple comparisons (Dunnet or Bennet) or *t* tests were performed to determine the significance of recorded values indicated in the figure legend. *p*-values of <0.05 were considered significant. Each value is presented as the mean ± standard error of the mean (SEM).

## 3 Results and Discussion

### 3.1 *A. sojae* Elicitation Alters Chromatograms of Pea Extracts

**Figure 1:**
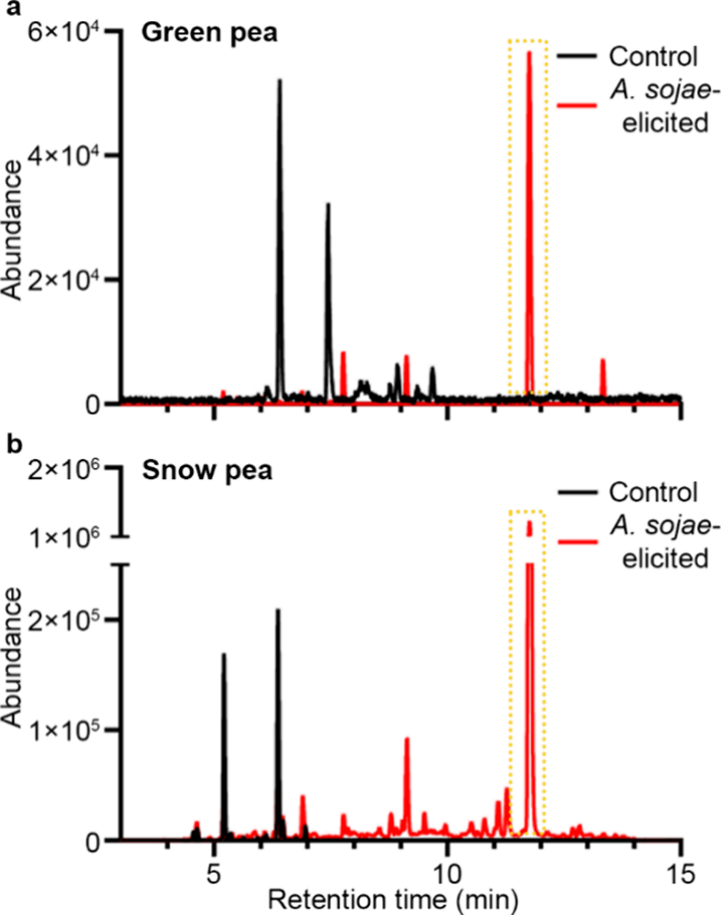
UPLC chromatograms of the methanol extracts of (a) control (black) and *A. sojae*-elicited (red) green pea and (b) control (black) and *A. sojae*-elicited (red) snow pea. The detection wavelength was set at 309 nm. (+)-Pisatin was identified at ∼11.5–12 min and indicated with a yellow dotted box.

Treatment of legume pods, with *A. sojae*, has previously been shown to elicit the production of the phytoalexin pisatin.^28−33,36^ Snow and green pea controls and *A. sojae*-treated extracts were evaluated by using UPLC-PDA, presented in Figure 1. Control extracts initially show that green pea’s major peaks (Figure 1a) are shifted further right and much in lower abundance (10^4^ vs 10^5^), compared to snow pea (Figure 1b). Once elicited, both show a sharp peak between 11.5–12, indicative of (+)-pisatin, with snow pea still having a much higher abundance, compared to green pea. Elicited extracts were also analyzed utilizing UPLC-MS and compared to nonelicited controls, presented in Figure S3. Elicitation altered chemical profiles of extracts, resulting in an increased number and intensity of peaks across the evaluated retention time. Significant changes in UPLC-MS chromatograms were localized between 9 and 12 min and corresponded to prior reports of pisatin production.^28−33,36^ Based on the observed changes in chromatograph profiles, cells were treated with pea extracts to determine effects on biological processes.

### 3.2 Treatment with Pea Extracts Alters the Transcriptional Activity of the Estrogen Receptor in MCF-7 and T47D Cells

**Figure 2:**
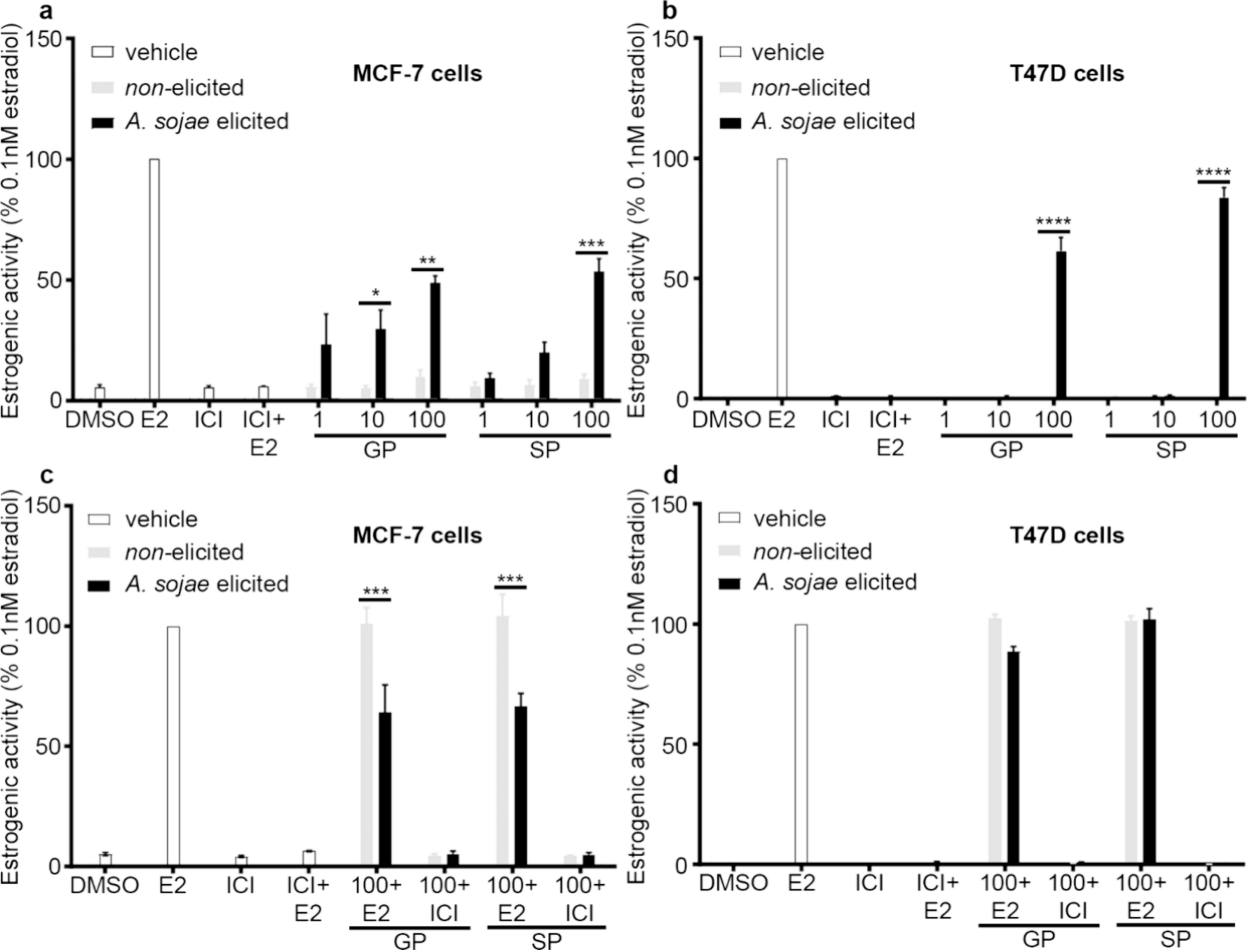
Effects of pea extracts on ERE transcriptional activity in MCF-7 (a,c) and T47D (b,d) cells. Cells were transiently transfected with the pGL2-ERE2x-TK-luciferase plasmid. After a 6 h transfection, cells were treated with increasing concentrations of nonelicited and *A. sojae*-elicited snow (SP) or green (GP) pea extracts (μg/mL, alone or combined with ICI or E2). The estrogenic activity was normalized to that of the positive control (0.1 nM E2). Data represent means ± SEM of three independent experiments. Two-way ANOVA and Šídák’s multiple comparison tests, comparing nonelicited to *A. sojae*-elicited extracts. *p < 0.05, **p < 0.01, ***p < 0.001, and ****p < 0.0001.

The estrogenic activities of snow (SP) and green pea (GP) extracts at various concentrations (1–100 μg/mL) were analyzed by measuring the transcriptional activation using a reporter gene assay and are presented in Figure 2a,b. All nonelicited extracts exhibited low estrogenic activity when applied to MCF-7 (<15%) or T47D (<2%) cells. The activity did not change significantly at higher extract concentrations. Treatment of MCF-7 cells with elicited GP or SP extracts (100 μg/mL) significantly increased estrogenic activity by 39 and 44%, respectively, compared to nonelicited controls (Figure 1a,b). Under the same conditions, the ERE transcriptional activity increased in T47D cells, suggesting that the effects are not cell line-specific.

Transcriptional activation of the estrogen receptor by legume extracts is a well-characterized phenomenon.^1^ The ability of nonelicited and *A. sojae*-elicited SP and GP extracts to increase the expression of an ERE-LUC reporter gene in MCF-7 and T47D cells suggests that there may be estrogenic activity, at least in part, via an ER-dependent mechanism. There is sparse information on the identification of flavonoids in SP; however, previous research has identified genistein, daidzein, kaempferol, and apigenin in green and yellow pea.^27^ The relatively mild ER agonist activity detected with both nonelicited and elicited SP extracts may be due to constitutive isoflavone or flavonoid components. However, the moderate antagonist activity observed after treatment of both SP and GP, with *A. sojae*, suggests enhanced antiestrogenic activity when compared with the untreated extract.

To determine if the extracts had antiestrogenic activity, cells were treated with E2 (0.1 nM) and the highest extract concentration (100 μg/mL). No antiestrogenic activity was observed in the nonelicited GP or SP extracts for both cell lines (Figure 2c,d). Treatment of MCF-7 cells with *A. sojae*-elicited GP or SP resulted in antiestrogenic activity (*p* < 0.001), evidenced by reduction of E2-induced estrogenic activity by 36% (GP) and 38% (SP) compared to cells treated with nonelicited extracts. In contrast, levels of estrogenic activity were comparable between T47D cells treated with nonelicited and elicited GP and SP. Combination treatment with ICI resulted in near-zero activity across all extracts, concentrations, and elicitation (<4% for MCF-7 and <1% for T47D cells).

The antiestrogenic activity observed using the *A. sojae*-elicited pea extracts suggests that an inducible component or phytoalexin contributes specifically to this antagonistic activity. The ability of legume extracts to antagonize ER gene transcription has been observed in *A. sojae*-elicited soybean extracts and phytoalexin components; glyceollins were identified as the antiestrogens.^16^ A similar phytoalexin component of SP and GP extracts, (+)-pisatin, may contribute to the antagonistic activity observed. We then evaluated whether changes in estrogenic activity impact cell proliferation and growth.

### 3.3 Elicited Pea Extracts Synergistically Enhance Proliferation in MCF-7 Cells

**Figure 3:**
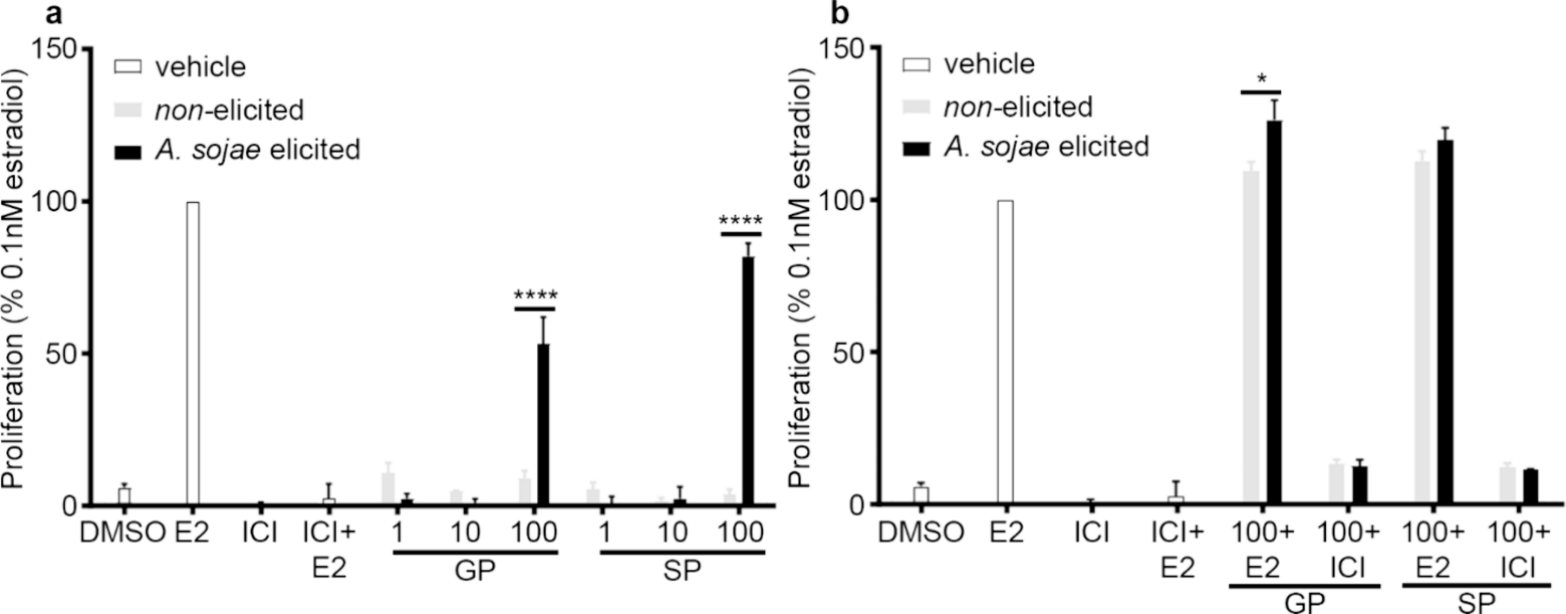
Effects of pea extracts on the proliferation in MCF-7 cells. Cells were transiently transfected with the pGL2-ERE2x-TK-luciferase plasmid. After a 6 h transfection, cells were treated with increasing concentrations of nonelicited and *A. sojae*-elicited snow (SP) or green (GP) pea extracts (alone (a) or combined with ICI or E2 (b)). Proliferation was evaluated using Alamar Blue and normalized to the positive control (0.1 nM E2). Data represent means ± SEM of three independent experiments. Two-way ANOVA and Šídák’s multiple comparison tests, comparing nonelicited to *A. sojae*-elicited extracts. *p < 0.05 and ****p < 0.0001.

MCF-7 cells were treated with nonelicited and elicited pea extracts, and cell proliferation was assessed. While treatment with lower concentrations did not affect cell proliferation, at 100 μg/mL, both elicited GP (53%) and SP (82%) extracts significantly increased proliferation (*p* < 0.0001) compared to nonelicited extracts (Figure 3a). These results are consistent overall with the effects on estrogenic activity observed (Figure 2a).

To evaluate potential antagonistic effects on proliferation, GP and SP extracts were combined with E2 or ICI (Figure 3b). All pea extract–E2 combinations (>110%) were higher than E2 alone, with the elicited GP extract being significantly higher (*p* < 0.05) than the nonelicited GP extract. Combinations of extracts with ICI exhibited an increase in proliferation (11–14%), compared to ICI alone (<1%). While estrogenic activity presented antiestrogenic properties of GP and SP extracts, proliferation maintained synergistic activity, under similar conditions. Since elicited pea extracts significantly altered estrogenic activity and proliferation, estrogen-mediated gene expression was evaluated to determine if gene expression aligned with the changes in cell activity. Moving forward, GP extracts were primarily used due to their ease of use, handling, and lower associated costs.

### 3.4 Green Pea Extracts Modulate Expression of Typical Estrogen-Mediated Genes

**Figure 4:**
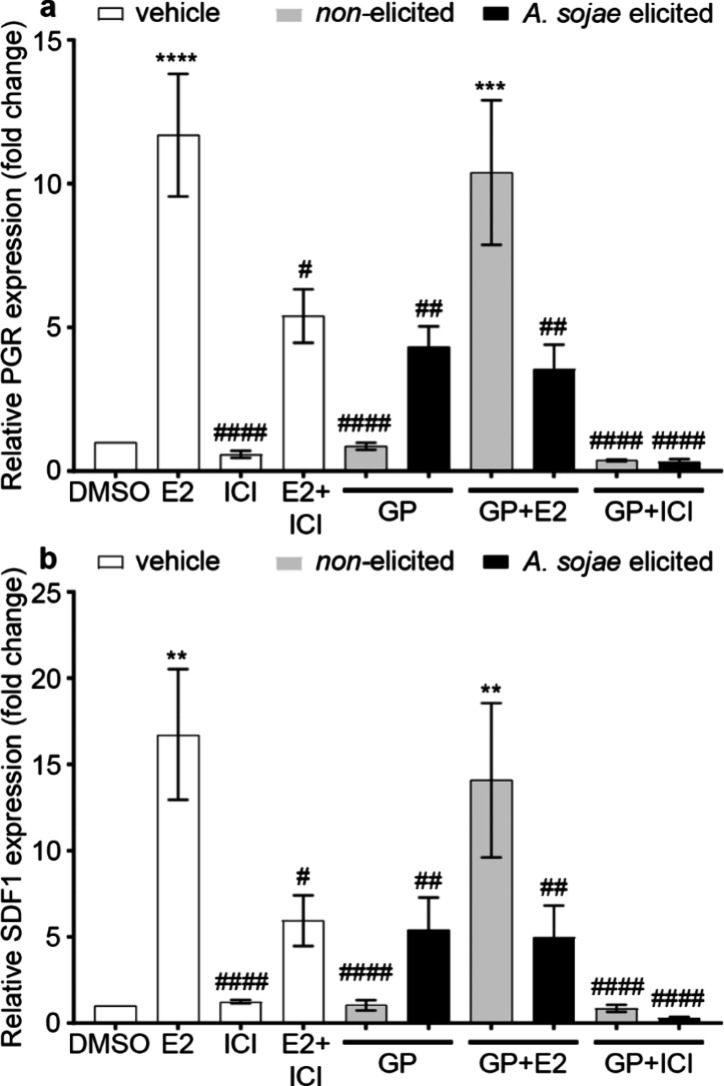
Effects of nonelicited and *A. sojae*-elicited pea extracts on PGR (PR) (a) and SDF1 (CXCL12) (b) expression in MCF-7 cells. Cells were grown in 5% DCC stripped media for 48 h prior to treatment for 24 h. At the end point, cells were collected, total RNA extracted, and cDNA synthesized. qRT-PCR was performed for ER response genes PGR and SDF1. Data were normalized to the DMSO control and housekeeping gene RPL13a (mean ± SEM, *n* = 3, with three biological replicates). One-way ANOVA and Dunnet’s multiple comparison tests, compared to DMSO (**p < 0.01, ***p < 0.001, and ****p < 0.0001) or E2 (^#^*p* < 0.05, ^##^*p* < 0.01, and ^####^*p* < 0.0001).

Progesterone receptor (PGR or PR) and stromal cell-derived factor 1 (SDF1 or CXCL12) gene expression was evaluated for MCF-7 cells treated for 24 h with vehicle controls and nonelicited and elicited GP extracts (10 μM). PGR expression significantly increased in MCF-7 cells treated with E2 (*p* < 0.0001) or nonelicited GP extract + E2 (*p* < 0.001), when compared to DMSO (Figure 4a). Similarly, SDF1 expression significantly increased in MCF-7 cells treated with E2 (*p* < 0.01) or nonelicited GP extract + E2 (*p* < 0.01), when compared to DMSO (Figure 4b). All other combinations showed no significant changes in gene expression. To evaluate antiestrogenic or antagonistic activity, the fold change was also compared to E2. PGR and SDF1 expression for ICI, nonelicited GP, and nonelicited and elicited GP + ICI was significantly lower (*p* < 0.0001). E2 + ICI (*p* < 0.05) and elicited GP and GP + E2 (*p* < 0.01) exhibited higher expression and were not as significantly different, when compared to E2.

Normalization of PGR and SDF1 fold changes, to E2, further elucidated the estrogenic or antiestrogenic activity. Elicitation of GP extracts resulted in an increase from ∼7 to 31% (SDF1) and 37% (SDF1). Cells drugged with nonelicited extracts and E2 were higher with 87% (PGR) and 83% (SDF1), but elicitation significantly reduced both PGR (30%) and SDF1 (29%) expression. Cells treated with ICI alone or in combination with extracts exhibited much lower expression (<8%).

Based on the changes in cell activity and estrogen-mediated gene expression, elicited GP extracts were further purified to isolate a primary elicitor of estrogenic changes. UPLC data, Figure 1, and prior literature indicated the presence of (+)-pisatin potentially providing the observed activity. A previous report has noted the use of *A. sojae* elicitation, in other legumes, leading to the increase in phytoalexins, with (+)-pisatin being a potential target.^16^

### 3.5 Isolation and Characterization of (+)-Pisatin Are Dependent on Feedstock

Peas are a source of many polyphenols and the constitutive flavonoids daidzein, genistein, kaempferol, and apigenin.^25^ Daidzein and genistein are found in many legumes including soybean, and both isoflavones have estrogenic activities.^2−4^ Further research has led to the discovery of inducible isoflavones in soybean including glycinol with estrogenic activity^59^ and glyceollin with antiestrogenic activity.^18−20^ (+)-Pisatin accumulates in high concentrations for peas under stress or elicitor treatment. There is potential for higher levels to be present in stressed or cut peas. Additionally, little is known about the hormonal effects of (+)-pisatin in mammalian systems. (+)-Pisatin was successfully identified using UPLC combined with electrospray ionization and quadrupole time-of-flight mass spectrometry (Q-TOF-MS). The positive ion mass spectra for each phytoalexin (M + H)^+^ were (+)-pisatin at *m*/*z* 315. The retention time, UV–vis spectrum, and low-energy (MS) and high-energy spectra of (+)-pisatin in SP and GP matched those of the pisatin standard. (+)-Pisatin displayed a molecular ion at *m*/*z* 315.0851 (M + H)^+^ with the (M–H_2_O + H)^+^ ion at *m*/*z* 297.0859 as the most intense ion in the MS spectrum. The high-energy fragment spectrum (MS^E^) of (+)-pisatin displayed intense product ions at *m*/*z* 295.0597, *m*/*z* 252.0414, *m*/*z* 225.0539, and *m*/*z* 139.0535. Evaluation of the UV spectra for the peak corresponding to (+)-pisatin revealed identical spectra compared with that in earlier reports with UV_max_ for (+)-pisatin at 280 and 309 nm.^60,61^

The phytoalexin content in the elicitor treatment of SP and GP extract was conducted using HPLC. In treatments with *A. sojae*, (+)-pisatin was undetectable in nonelicited extracts and increased to ∼1.2 mg/g (SP) and 0.4 mg/g (GP), after elicitation (Figure S4). SP exhibited a significantly higher (*p* < 0.0001) (+)-pisatin content, when compared to GP. While elicited SP extracts produced more (+)-pisatin, SPs were more expensive and had to be used fresh, and while the pod material produced (+)-pisatin, it was much lower than the seeds. In contrast, GPs were less expensive and dried off-the-shelf products. They only required a short soak before cutting. Moving forward, (+)-pisatin isolated from GP was used for subsequent studies.

### 3.6 (+)-Pisatin Treatment Exhibits Estrogenic Activity in MCF-7 and T47D Cells

**Figure 5:**
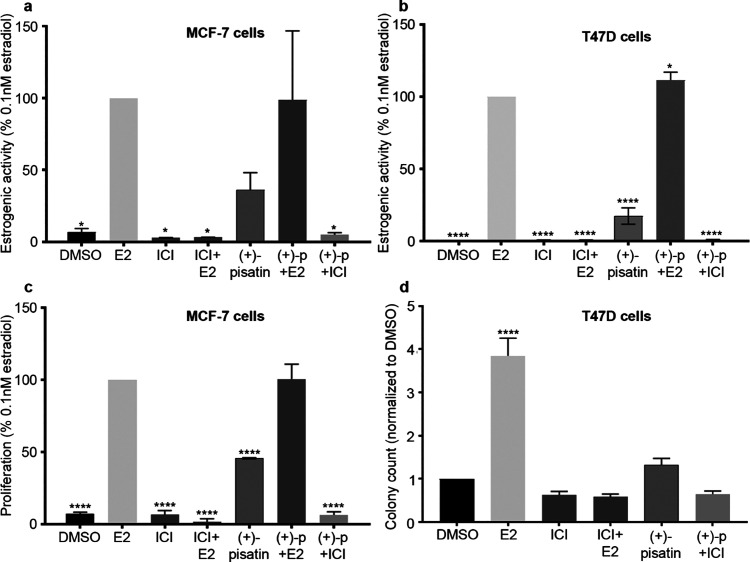
Effects of (+)-pisatin (p) on ERE transcriptional activity (a,b), proliferation (c), and colony formation (d) in MCF-7 and T47D cells. Cells were treated with (+)-pisatin (alone or in combination with ICI or E2). The estrogenic activity was normalized to that of the positive control (0.1 nM E2 or DMSO). Data represent means ± SEM of three independent experiments. One-way ANOVA and Dunnet’s multiple comparison tests, compared to E2 (a–c) or DMSO (d). *p < 0.05, ****p < 0.001, and ****p < 0.0001.

The proliferation of ER^+^ cancer cells is a well-established biological response to E2 and a useful screening tool for compounds that may function as estrogen agonists.^40,62^ Additionally, the ability of (+)-pisatin to stimulate the growth of breast cancer cells and display estrogenic activity has been reported previously.^38^ Studies with MCF-7 cells (Figure 5a,c) showed (+)-pisatin-induced estrogenic activity and proliferation. MCF-7 cells treated with (+)-pisatin exhibited 37% estrogenic activity, normalized to E2, while DMSO controls were 7%. Treatment with (+)-pisatin and E2 showed no signs of antiestrogenic activity (99%); the large standard error would require further investigation. One-way ANOVA analysis determined that (+)-pisatin and (+)-pisatin + E2 were not different from E2 treatment alone. All other conditions were significantly different (*p* < 0.05). MCF-7 proliferation presented similar results (Figure 5c), with (+)-pisatin treatment having 46 and 100% for (+)-pisatin + E2. Conversely, all conditions were significantly different (*p* < 0.0001), compared to E2, except (+)-pisatin + E2. In both estrogenic activity and proliferation, neither show the antiestrogenic activity presented previously in the pea extracts (Figures 2 and 3).

Mirroring studies in MCF-7 cells, T47D cells were treated and evaluated for estrogenic activity and colony formation (Figure 5b,d). The results were similar, with only (+)-pisatin + E2 treatment exhibiting a significant increase in activity (*p* < 0.05) and all other conditions reporting a significant decrease (*p* < 0.0001), Figure 5b. Colony counting, an evaluation method for proliferation, of treated cells only showed E2 treatment significantly increasing (*p* < 0.0001), with all other treatments not different from the DMSO control (Figure 5d).

(+)-Pisatin is primarily derived from 2,7,4′-trihydroxyisoflavonone during biosynthesis^29^ and is organized in a pterocarpan structure. Produced in the isoflavonoid pathway, it contains rings ABCD and exists in nature in two stereoisomeric forms.^28^ (+)-Pisatin, the (+) stereoisomer, is unique among flavonoid phytoalexins since most legumes produce the (−) stereoisomer.^63^ With a conformation similar to a related phytoalexin, phaseollin,^21^ both have displayed moderate estrogenic activity and antiestrogenic activity.^21^ The ability of (+)-pisatin to induce MCF-7 cell proliferation correlates well with the activation of ER transcriptional activity, and this correlation points to (+)-pisatin acting through an estrogen receptor (ER)-dependent mechanism. To confirm the mechanism, ER-mediated pathways were evaluated using qRT-PCR and Western blotting.

### 3.7 (+)-Pisatin Regulates Expression of Estrogen-Mediated Genes after Treatment in MCF-7 and T47D Cells

**Figure 6:**
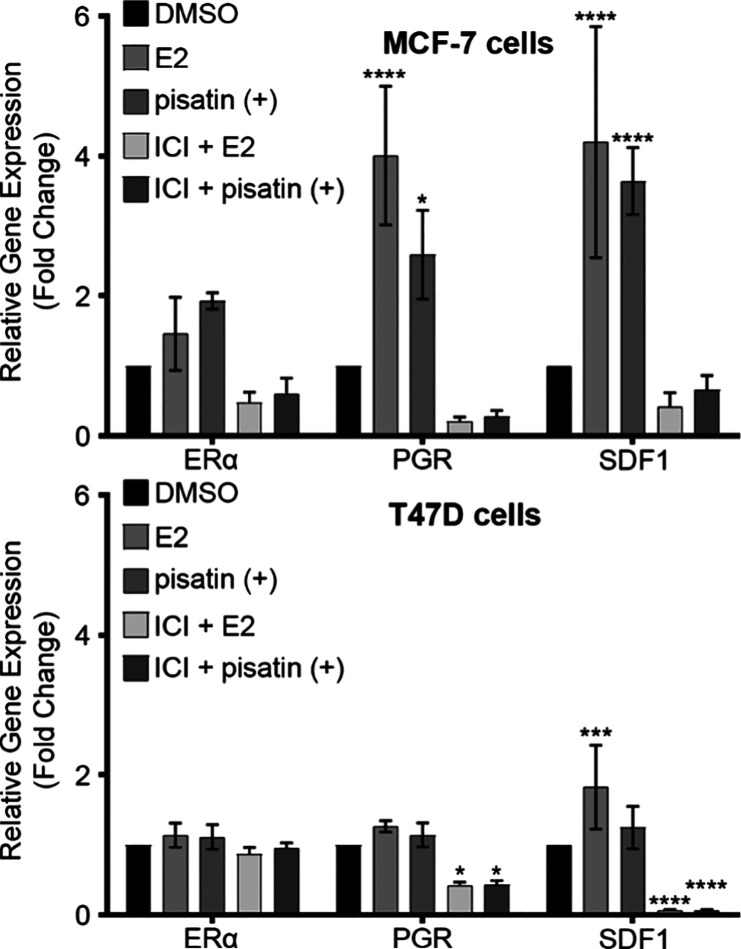
Effects of (+)-pisatin on ERα, PGR, and SDF1 expression in MCF-7 and T47D cells. Cells were grown in 5% DCC stripped media for 48 h prior to stimulation with (+)-pisatin for 24 h. At the end point, cells were collected, total RNA extracted, and cDNA synthesized. qRT-PCR was performed for ERα and ER response genes PGR and SDF1. Data were normalized to the DMSO control and housekeeping gene RPL13a (mean ± SEM, *n* = 3, with three biological replicates). Two-way ANOVA and Šídák’s multiple comparison tests, compared to DMSO. *p < 0.05, ***p < 0.001, and ****p < 0.0001.

Gene expression for estrogen receptor alpha (ERα), progesterone receptor (PGR), and stromal cell-derived factor 1 (SDF1) has been used in prior works for evaluating novel plant-based compounds.^64,65^ These genes are associated with cell proliferation and/or tumorigenesis, making them ideal targets for evaluating ER regulation in ER^+^ cell lines. Furthermore, an estrogen receptor-degrading agent, ICI, was used in conjugation with (+)-pisatin and E2 to determine the mechanism of action.^66^ Based on the prior studies (not presented here), the tested concentration of (+)-pisatin (10 μM) was used to evaluate fold changes in estrogen-mediated gene expression in MCF-7 and T47D cells (Figure 6). (+)-Pisatin addition significantly increased the expression of PGR (*p < 0.05) and SDF1 (****p < 0.0001), and E2 treatment also significantly increased PGR (****p < 0.0001) and SDF1 (****p < 0.0001) gene expression. These results correlate well with the luciferase studies showing that (+)-pisatin, at 10 μM, elicits an estrogenic/proliferative response in MCF-7 and T47D cells. The addition of ICI predrugging for both (+)-pisatin and E2 depleted gene expression for all evaluated genes to below the vehicle/negative (DMSO) control. Since ICI degrades cell surface estrogen receptors, the reduction of gene expression indicates that (+)-pisatin acts primarily through the estrogen receptor pathway. The (+)-pisatin mechanism of action mimics E2, as both exhibited similar responses when predosed with ICI. To further understand the mechanisms altered through (+)-pisatin treatment, a broad PCR array was used to broadly profile over 80 genes related to breast cancer pathways.

### 3.8 PCR Array of MCF-7 Cells after (+)-Pisatin Treatment

PCR arrays profile large sets of genes associated with specific diseases or pathways that aid in determining drug-related changes in a rapid and precise process and have been used in prior works.^67^ To determine whether (+)-pisatin could induce changes in gene expression of MCF-7 cells, a breast cancer pathway PCR array (angiogenesis, cell cycle, DNA damage and repair, cellular senescence, metabolism, apoptosis, EMT, telomeres and telomerase, and hypoxia signaling) was used (Table S2). Genes were normalized to MCF-7 cells with the vehicle control. There was no clear trend of the gene-associated pathways for upregulation, but more of the telomere and telomerase genes were among the most downregulated (TERF2IP and TNKS2). Of the 79 genes evaluated, four genes exhibited a >2-fold change in expression, MEK1, CCND3, and ERK1/2. Based on changes in ER-mediated gene expression, it was important to determine if these changes correlated with ERα protein levels.

### 3.9 Temporal Modulation of ERα Protein Expression with (+)-Pisatin Treatment

Ligand binding has been shown to promote ubiquitination and protein degradation to varying extents depending on the type of ligand.^68^ Binding of estrogen to the ERα quickly triggers the process of ERα ubiquitylation and degradation.^69,70^ Unbound ERα has high stability with a half-life of up to 5 days; however, upon binding to a ligand, the half-life of ERα significantly decreases to 3–5 h.^71−73^ The processes of ERα ubiquitination and proteasome activity are closely connected to the activation of ERα-dependent transcription. The interaction of a ligand triggers both the activation of ERα-dependent gene transcription and the process of ERα ubiquitination. Since our results demonstrated that (+)-pisatin induced proliferation of ERα-positive breast cancer cells in an ER-dependent manner and ERα-dependent gene transcription, we decided to determine the effect of (+)-pisatin on ERα protein in MCF-7 breast cancer cells.

**Figure 7:**
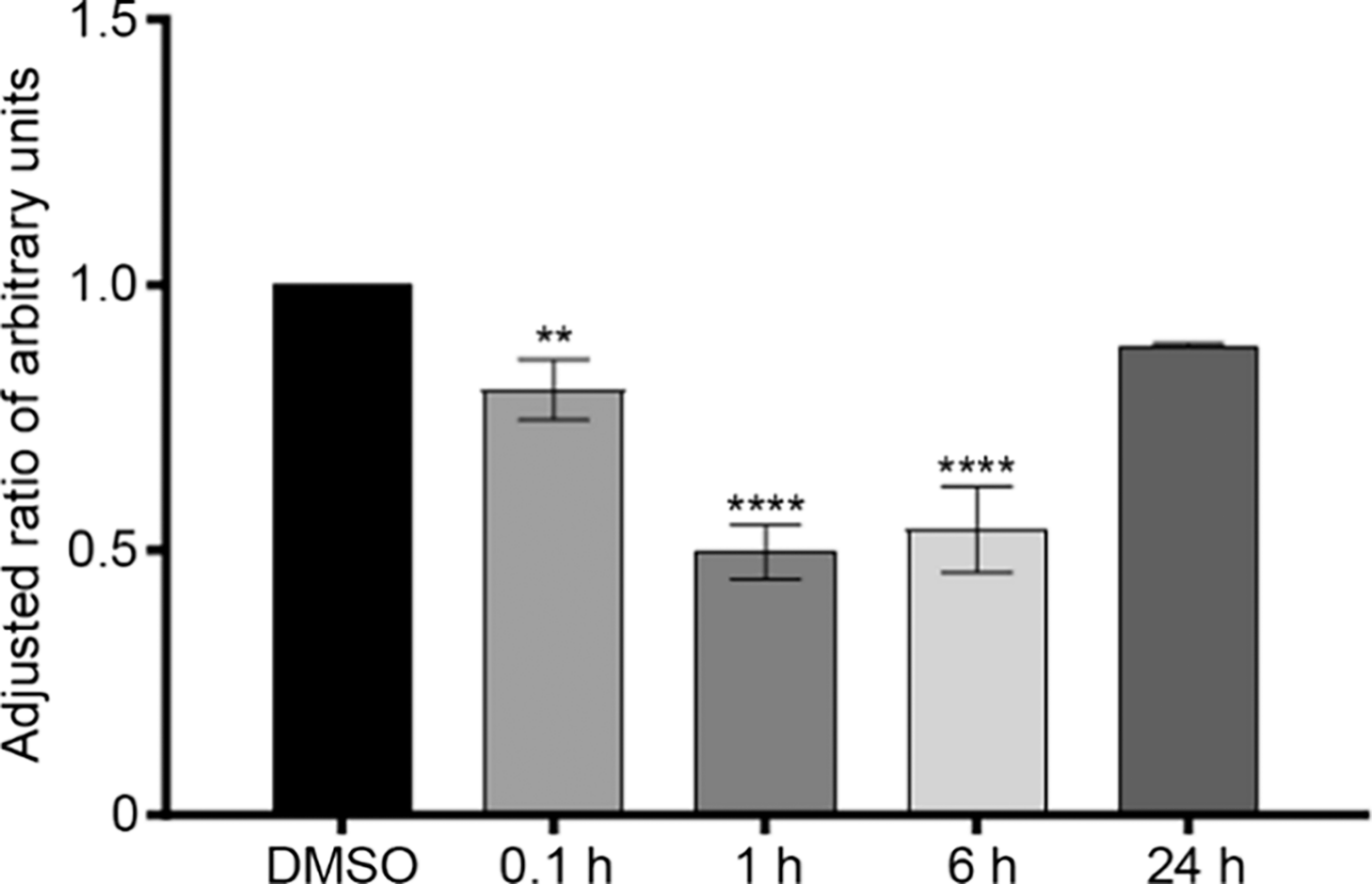
Modulation of the ERα protein expression is time-dependent. MCF-7 cells were grown until 70–80% confluency, media were aspirated, cells were washed with PBS three times, and cells were incubated in stripped media for 48 h. After 48 h, DMSO (2 μL) and (+)-pisatin (10 μM) were added and incubated at consecutive time points of 0, 1, 6, and 24 h. After each time point, media were aspirated, cells were washed with cold PBS and aspirated, and cell pellets were collected for protein extraction. Data were normalized to the DMSO control (mean ± SEM, *n* = 3, with three biological replicates). One-way ANOVA and Dunnett’s multiple comparison tests, compared to DMSO. **p < 0.01 and ****p < 0.0001.

MCF-7 cells, when treated with (+)-pisatin, demonstrated a gradual time-dependent reduction in ERα protein expression, as depicted in Figure 7. Jess blots are presented in Figure S4. Expression levels were dramatically lowered by 20% within approximately 0.1 h after treatment, as compared to the DMSO control (*p* < 0.01). The minimum level of ERα expression was observed 1 h after treatment, with ∼50% reduction. This level remained steady 6 h after (+)-pisatin treatment, with ∼47% reduction. However, 24 h post-treatment, ERα expression increased and was not significantly different from the DMSO control. The expression of ERα, assessed by a capillary-based Western assay (Jess), shows a strong correlation with previously documented data on estrogenic activity, proliferation, and gene expression. With a more complete understanding of the cellular mechanisms, molecular modeling and docking were used to visualize the interactions between compounds and the ER binding pocket.

### 3.10 (+)-Pisatin Docks to ERα in a Bent Conformation and Is Site-Dependent

Estradiol (E2) is present in Figure S3a binding with the ERα pocket, and 4OH-tamoxifen is shown binding to the antagonist ERα pocket (Figure S6b). The most favorable scoring poses of (+)-pisatin in 1ERE (estradiol, agonist) and 3ERT (4OH-tamoxifen, antagonist) pockets of ERα each involve a bent conformation of the pterocarpan ring system (Figure S6c,d). In both the agonist and antagonist ER pockets, one ring of the (+)-pisatin occupies the A ring or phenolic binding region of the ER without the traditional hydrogen bonding to Glu 353, Arg 394, and/or water of most estrogens. The docking pose of the bent (+)-pisatin in the 1ERE (estradiol, agonist) pocket utilizes a unique binding mode that occupies the “extra space” known to be present above estradiol in ER as well as a novel hydrogen bond between the lone hydroxyl of (+)-pisatin and Thr 347. The binding mode suggested by the docking of (+)-pisatin to the 3ERT (4OH-tamoxifen, antagonist) ER structure involves occupation of both the A ring binding region of the ER pocket as well as the antagonist pocket. The antagonist pocket of ER is formed by the aryl tertiary amine group of tamoxifen that directly displaces helix-12 to induce antagonist activity (Figure S6d). In an estrogen receptor binding assay, (+)-pisatin displayed ERβ binding slightly higher than that of the phytoalexin phaseollin (RBA = 0.530%) and had a higher ERα binding affinity when compared to phaseollin.^21^ Additionally, (+)-pisatin displayed lower binding to ERβ and ERα when compared to other more estrogenic constitutive isoflavones like genistein and daidzein from soybean^74^ and other legumes. This ability to bind the estrogen receptor was confirmed using docking simulations. Docking simulations in this study present reasonable potential binding modes for (+)-pisatin to interact with both the agonist and antagonist forms of the ER ligand pocket. Docking of (+)-pisatin to the ER agonist pocket presents a unique binding mode that occupies the “extra space” in the ER ligand pocket as well as stabilization by a novel hydrogen bond. These simulations are consistent with the weak but significant ER binding activity and are consistent with the partial agonist, partial antagonist activity observed.

Several studies have demonstrated that phytoestrogens exert their stimulatory effect on the ER by binding to the same site as E2 with some differences in ligand-binding specificity and transactivation between ERα and ERβ.^74−77^ Of particular interest was the observation that certain isoflavones may bind with higher affinity and possess higher agonistic activity toward ERβ.^74−77^ To assess the ability of (+)-pisatin to bind to ERα and ERβ, a competitive binding assay with fluorescence detection was utilized. Table S3 details the results for the competitive binding assay using ERα. (+)-Pisatin had relative binding affinities at 0.326% for ERα and 0.574% for ERβ (not presented here), normalized to the E2 control. RNA sequencing of treated MCF-7 cells was used to determine a wide range of alternative pathways, tissue systems, and biological processes.

### 3.11 (+)-Pisatin Treatment Induces an ER-Responsive Transcriptome

**Figure 8:**
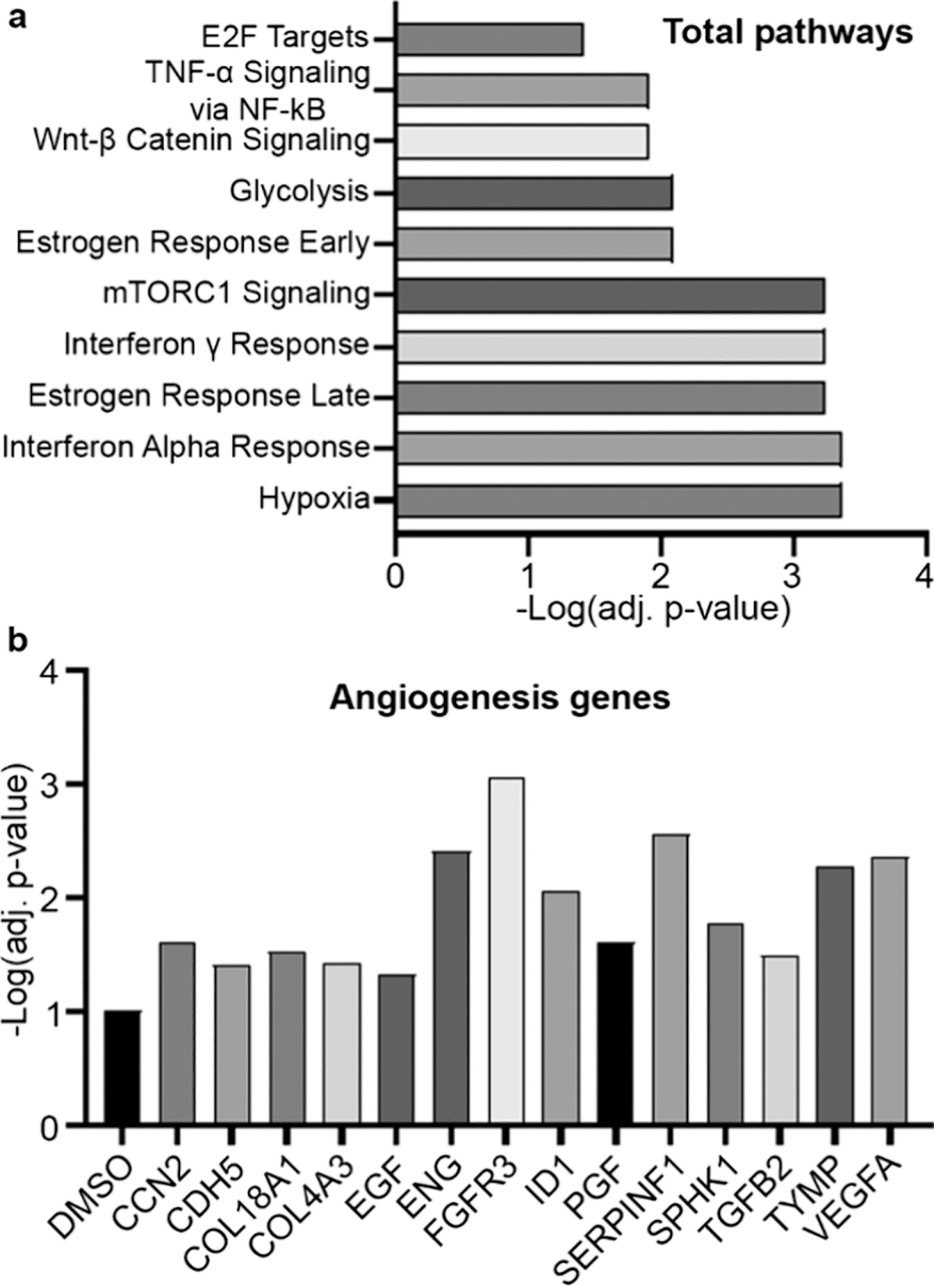
RNA sequencing of MCF-7 cells treated with (+)-pisatin, compared to untreated. (a) Top 10 total pathways showing that (+)-pisatin induces ER gene signatures and (b) expression of angiogenic genes showing that (+)-pisatin represses angiogenic factors.

To determine global effects of (+)-pisatin on cellular transcription, MCF-7 cells were treated for 24 h, and then, RNA sequencing was performed. (+)-Pisatin-treated MCF-7 cells had a total of 1970 genes changed with 593 genes demonstrating a significant increase in expression and 1377 genes demonstrating a significant decrease in expression. To determine gene set enrichments to known pathways, Enrichr and the MSigDB Hallmark gene set were used. Altered gene sets were associated with hypoxia and early and late estrogen response in addition to mTORC1 signaling pathways for all significantly changed genes. Gene enrichments for upregulated pathways were highly associated with mTORC1 signaling and cell proliferation processes, such as E2F targets and G2-M targets. Genes repressed by (+)-pisatin were associated with inflammatory responses such as interferon α and γ signaling and TNF-mediated responses (Figure 8a). Comprehensive pathway analysis changes are presented in Figure S7. In addition, there was a trend of (+)-pisatin-induced downregulation of angiogenesis-associated genes including CDH5, ENG, SERPINF1, TYMP, and VEGF-A (Figure 8b). Downregulation of these genes in tumor cells does not confirm functional inhibitory effects of (+)-pisatin on angiogenesis. We previously reported differential phytochemical effects on tumor cells and endothelial cells^78^ and therefore proceeded to quantify the effects of (+)-pisatin on endothelial cell proliferation and *in vitro* vasculogenesis.

### 3.12 (+)-Pisatin Enhances Bulk Tissue Vasculogenesis and Promotes Cell Cycle Exit

**Figure 9:**
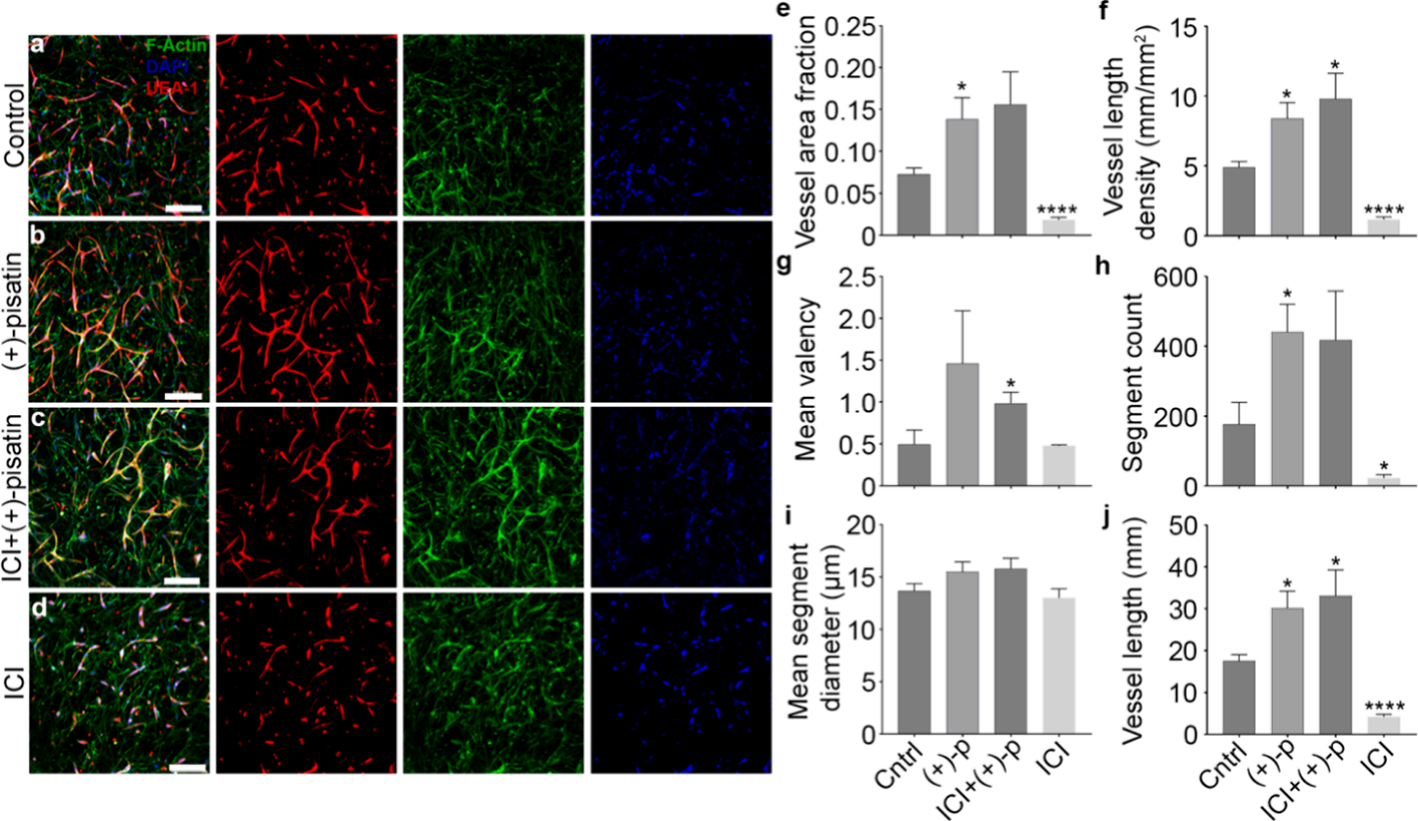
Provasculogenic effects of (+)-pisatin in engineered bulk tissue vasculogenesis. (a–d) Representative 3D laser scanning confocal microscopy (LSCM) images of the microvascular network in the bulk tissue after 6 days in culture treated with 10 μM (+)-pisatin, 10 μM (+)-pisatin + 1 μM ICI, and 1 μM ICI only; control groups are treated with equal volumes of DMSO. Endothelial cells are labeled with UEA-1 (red). F-actin in all cells is labeled with phalloidins (green), and fibroblasts are green only. Nuclei of all cells are labeled with DAPI (blue). Scale bar = 250 μm. (e–j) Morphometric analysis of resultant vessels after treatment with (+)-pisatin (*n* = 3). Vessel coverage (e,f), complexity (g,h), and size dimensions (i,j). Statistical analyses were performed using an unpaired *t* test with Welch’s correction. Comparisons are with vehicle groups (*p < 0.05, **p < 0.01, ***p < 0.001, and ****p < 0.0001). Error bars represent SEM.

Tissue-scale functional effects of (+)-pisatin on human endothelial cells were assessed using a microphysiological model of bulk tissue vasculogenesis previously used as an assay to investigate phytochemical effects.^78^ A polydimethylsiloxane (PDMS) device was used to constrain 3D vascularized tissues composed of female HUVEC and female (XX) human lung fibroblasts (HLF) embedded in fibrin and collagen type I hydrogel. Ten μM (+)-pisatin qualitatively enhanced vascular network assembly and organization relative to control tissues maintained with a standard VEGF-A containing medium after 6 days of culture (Figure 9a,b). Previous studies using the same vasculogenesis assay configuration measured similar effects with exogenous estradiol,^56^ thereby supporting the notion that (+)-pisatin acts in part as an estrogen receptor agonist. To explore this notion, we tested the effect of fulvestrant (ICI) on vasculogenesis with and without (+)-pisatin (Figure 9c,d). ICI alone potently inhibited vasculogenesis at a concentration of 1 μM, indicating that vasculogenesis is dependent on estrogen receptor activation. However, ICI did not significantly inhibit vasculogenesis in the presence of 10 μM (+)-pisatin, suggesting that (+)-pisatin may have a higher estrogen receptor binding affinity than ICI. It is possible that (+)-pisatin stimulates vasculogenesis via nonestrogen-mediated mechanisms, but the finding that ICI alone is potently inhibitory suggests that (+)-pisatin competes with ICI for estrogen receptor occupancy and generates enough agonistic activation to preserve vasculogenesis. Morphometric analyses revealed that (+)-pisatin significantly increased the average vessel area fractions, vessel length densities, vessel segment counts, and total vessel lengths (Figure 9e,f,h,j). Mean network valency exhibited a similar nonsignificant increasing trend, while there was no apparent effect on vessel diameters (Figure 9g,i). These findings suggest an accelerated trajectory of network assembly and maturation driven by (+)-pisatin, which involves a switch from activation to quiescence in endothelial cells following the structural organization of the vasculature. While the estrogen receptor binding affinity of (+)-pisatin is unknown, these data suggest that it is a strong agonist that outcompetes ICI for receptor occupancy.

Effects of (+)-pisatin on the endothelial cell cycle status in 2D expansion cultures were measured to investigate potential actions on the balance between activation and quiescence (Figure S8). (+)-Pisatin decreased the Ki67 proliferation index in a dose-dependent manner, but only 25 μM produced a significant reduction (Figure S8b). Total cell numbers were similar across groups despite the trend of a decreasing Ki67 index, suggesting a subtle shift toward cell cycle exit rather than distinct pharmacological inhibition of cell proliferation (Figure S8c). Taken together, assays of vasculogenesis and cell cycle status suggest that (+)-pisatin can both enhance vascular morphogenesis and promote endothelial cell quiescence. Thus, (+)-pisatin and phytochemicals with similar modes of action may positively impact wound healing and tissue regeneration while curtailing excessive proliferation associated with pathological neovascularization.

## Supporting Information Available

(Figure S1) MS spectrum of (+)-pisatin, (Figure S2) MS–MS spectrum of (+)-pisatin, (Figure S3) UPLC-ESI-MS chromatograms (TIC) of pea extracts, (Figure S4) (+)-pisatin content (mg/g) from *A. sojae*-elicited green and snow pea extracts, (Table S1) primer sequences used for PCR, (Table S2) breast cancer pathway PCR array gene changes compared to the DMSO control, (Figure S5) ERα Jess blot, (Figure S6) (+)-pisatin docking into the ERα pocket and the effect on estrogenic activity, (Table S3) relative binding affinity of (+)-pisatin to the ERα receptor, (Figure S7) comprehensive pathway analysis changes, and (Figure S8) dose–response of human umbilical vein endothelial cells after treatment with (+)-pisatin (PDF)

## Abbreviations

E2: 17β-estradiol
cDNA: complementary DNA
DI: deionized H_2_O
DCC: dextran-coated charcoal-treated
DMSO: dimethyl sulfoxide
DNB: DNA nanoball
DMEM: Dulbecco’s modified Eagle’s medium
ER: estrogen receptor
ERE-LUC: estrogen receptor response element luciferase
ECM: extracellular matrix
FBS: fetal bovine serum
ICI: fulvestrant
GP: green pea
MSE: high-energy mass spectrometry
HLF: human lung fibroblasts
HUVEC: human umbilical vein endothelial cells
IPA: isopropyl alcohol
LSCM: laser scanning confocal microscope
MS: mass spectrometry
PFA: paraformaldehyde
PDMS: polydimethylsiloxane
PDA: polydopamine
PGR: progesterone receptor
SP: snow pea
SEM: standard error of the mean
SDF1: stromal cell-derived factor 1
UEA-1: Ulex europaeus agglutinin I
UPLC-ESI-MS: ultraperformance liquid chromatography electrospray ionization tandem mass spectrometry
UDG: uracil-DNA-glycosylase
