# Supplementary material for: Context-Dependent Estrogenic Actions of (+)-Pisatin Produced in Elicited Green or Snow Pea (Pisum sativum)
Source: J Agric Food Chem. 2024 Dec 12;72(51):28255–69. doi: 10.1021/acs.jafc.4c06409 (PMC11674159; doi:10.1021/acs.jafc.4c06409)
Supplement: Supplementary file 1 — jf4c06409_si_001.pdf [file jf4c06409_si_001.pdf]

Supporting Information

Context-dependent Estrogenic Actions of (+)-Pisatin Produced in Elicited Green or Snow Pea (*Pisum Sativum*).

Jorge A. Belgodere[1,2,3], Megan C. Benz[1], G. Wills Kpeli[4], Jack R. Elliott[1], Steven Elliott[1], Jack D. North[1], Isaac J. Ponder[2], Peng Ma[5], Sophie R. Dietrich[1], Thomas Cheng[1], Khoa Nguyen[1], Syreeta L. Tilghman[6], John A. McLachlan[1], Binghao Zou[7], Muralidharan Anbalagan[7], Brian Rowan[7], Mark Mondrinos[4], Thomas E. Wiese[5], Van T. Hoang[1,3], Bridgette M. Collins-Burow[1,3], Elizabeth C. Martin[1,3], Matthew E. Burow[1,3*], Stephen M. Boué[8*]

1 Tulane Department of Medicine, Section of Hematology & Medical Oncology, Tulane University Health Science Center, New Orleans, LA 70112

2 Department of Biological and Agricultural Engineering, Louisiana State University and Agricultural Center, Baton Rouge, Louisiana 70803

3 Tulane Cancer Center, Tulane University, New Orleans, LA 70112

4 Department of Biomedical Engineering, Tulane University, New Orleans, LA 70112

5 Xavier University School of Pharmacy, Xavier University, New Orleans, LA 70125

6 Pharmaceutical Sciences Division, College of Pharmacy and Pharmaceutical Sciences, Florida A&M University, Tallahassee, FL 32307

7 Department of Structural and Cellular Biology, Tulane University School of Medicine, New Orleans, LA 70112

8 U. S. Department of Agriculture, Agricultural Research Service, Southern Regional Research Center, New Orleans, LA 70179

KEYWORDS: legume; snow pea; green pea; phytoalexin; estrogenic; anti-estrogenic; (+)-pisatin

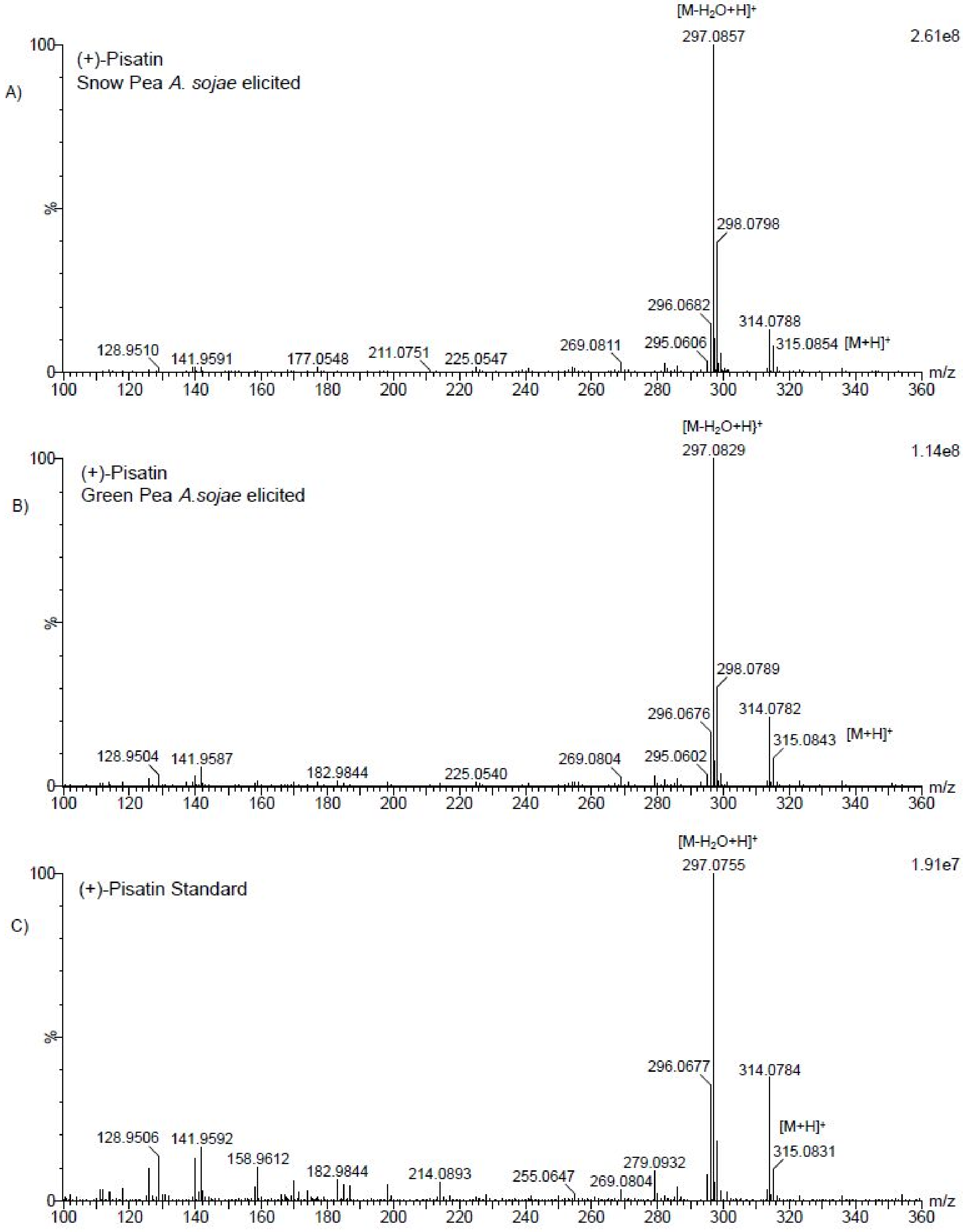

Figure S1. Electrospray high resolution MS spectrum of (+)-pisatin from (a) *A. sojae*-elicited snow pea; (b) *A. sojae*-elicited green pea; and (c) standard.

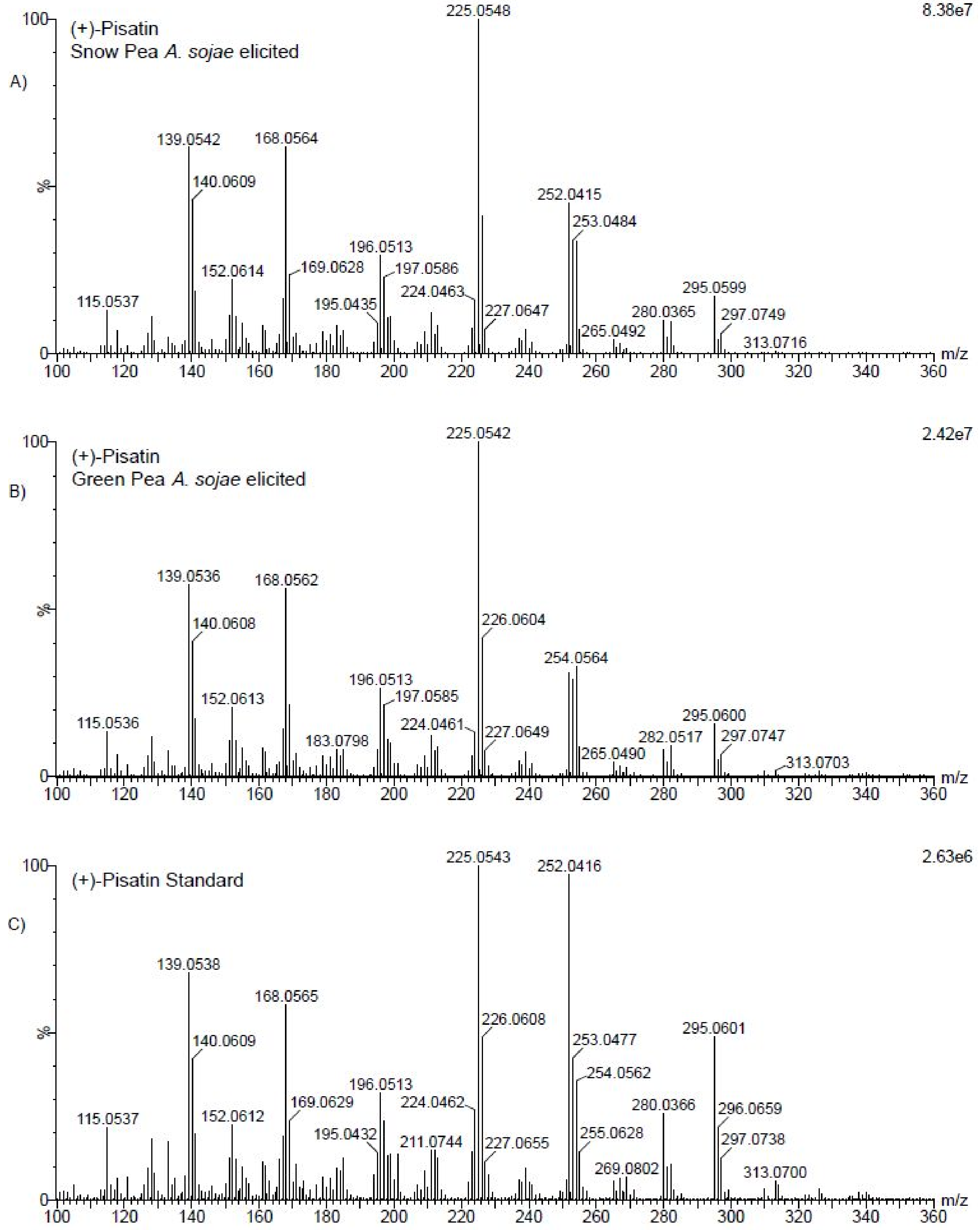

Figure S2. Electrospray high resolution MS-MS Spectrum of (+)-pisatin from (a) *A. sojae*-elicited snow pea; (b) *A. sojae*-elicited green pea; and (c) standard.

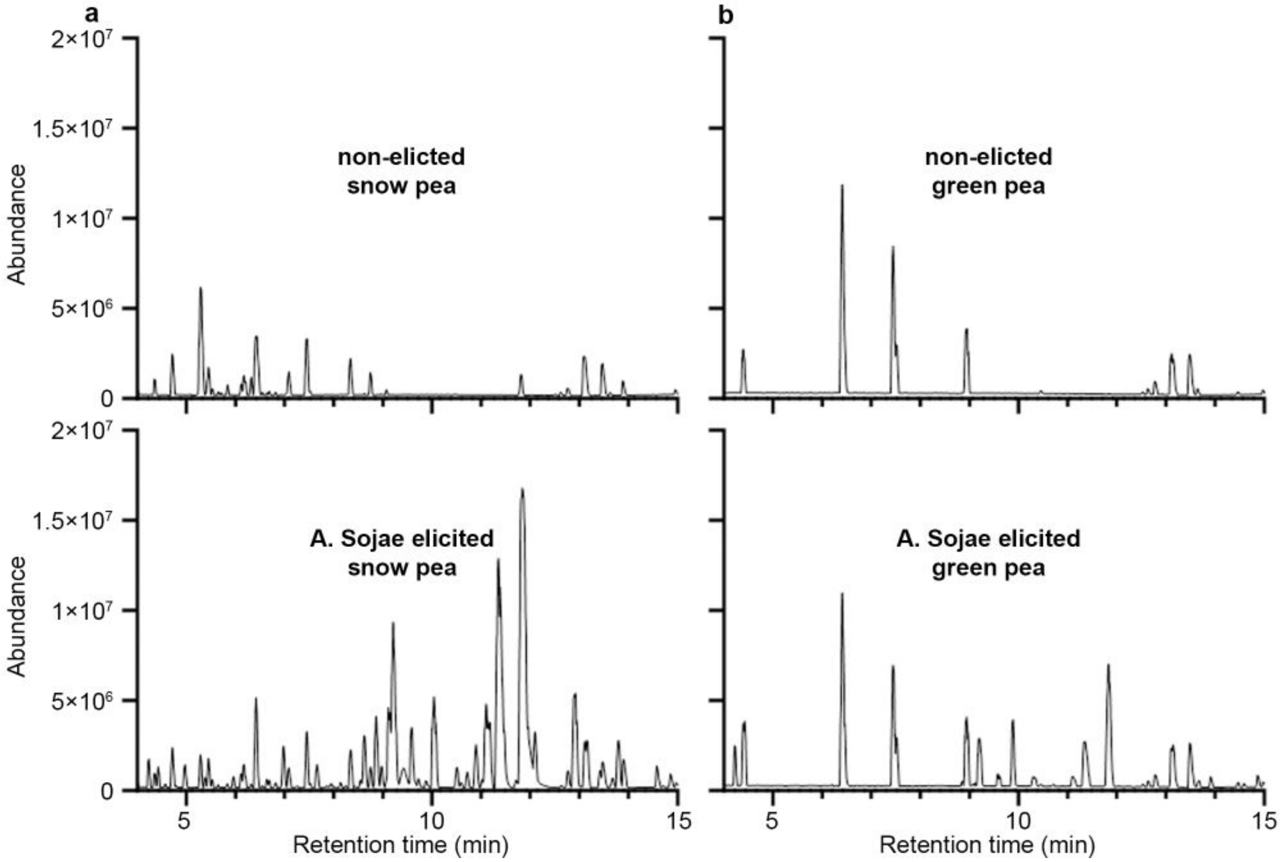

Figure S3. UPLC-ESI-MS chromatograms (TIC) of the methanol extracts of a) non-elicited (top) and *A. sojae* elicited (bottom) green peas and b) non-elicited (top) and *A. sojae* elicited (bottom) snow peas.

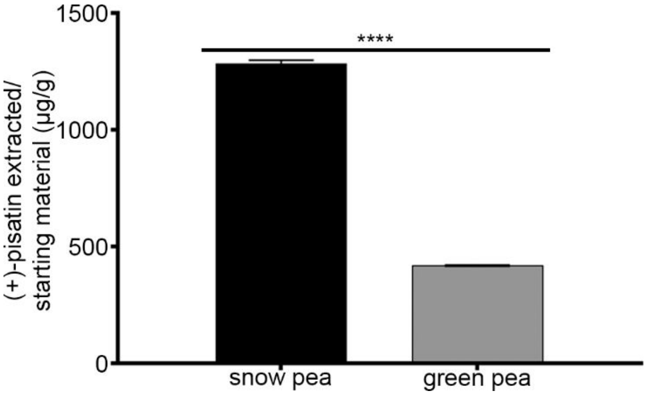

Figure S4. HPLC evaluation of (+)-pisatin extracted from *A. sojae* elicited green and snow pea extracts. Data represents mean ± SEM of 3 independent experiments. Unpaired t-test, ****p<0.0001.

Table S1: Primer sequences used for PCR

Gene	Forward	Reverse
B2M	AGATGAGTATGCCTGCCGTG	ACATGTCTCGATCCCACTTAAC
RPL13a	GAGGCCCCTACCACTTCC	AACACCTTGAGACGGTCCAG
ERα	GGCATGGTGGAGATCTTCGAA	CCTCTCCCTGCAGATTCATCA
PGR	TACCCGCCCTATCTCAACTACC	TGCTTCATCCCCACAGATTAAACA
SDF1	ACACTCCAAACTGTGCCCTTCA	CCACGTCTTTGCCCTTTCATC

Table S2: Breast cancer pathway PCR array gene changes, compared to DMSO control.

Gene	Fold-change	Gene	Fold-change	Gene	Fold-change
FLT1	2.44	MAP2K3	0.99	OCLN	0.74
CCND3	2.22	MAPK14	0.95	PGF	0.73
ERCC3	2.19	G6PD	0.94	CASP9	0.72
MKI67	1.74	DSP	0.93	ANGPT2	0.72
MAP2K1	1.62	CCND2	0.92	CCL2	0.69
BCL2L11	1.41	SERPINB2	0.91	ETS2	0.68
CPT2	1.37	TNKS	0.90	SKP2	0.67
UQCRFS1	1.37	APAF1	0.89	TBX2	0.66
CASP7	1.37	WEE1	0.89	FGF2	0.62
KDR	1.28	COX5A	0.87	CDC20	0.62
CDH2	1.20	BIRC3	0.86	TEK	0.59
NOL3	1.20	POLB	0.86	ERCC5	0.59
IGFBP3	1.15	ATP5A1	0.86	TERF1	0.55
TEP1	1.15	LPL	0.82	GSC	0.52
SLC2A1	1.13	SNAI2	0.82	EPO	0.52
CA9	1.12	MCM2	0.82	ACLY	0.52
SERPINF1	1.12	FOXC2	0.82	PINX1	0.50
LDHA	1.09	XIAP	0.81	SNAI1	0.50
GPD2	1.09	BMI1	0.80	IGFBP7	0.49
IGFBP5	1.08	SOX10	0.79	AURKA	0.49
GADD45G	1.05	VEGFC	0.79	DDB2	0.48
ARNT	1.05	SNAI3	0.78	TNKS2	0.45
ANGPT1	1.05	CFLAR	0.77	TERF2IP	0.29
CASP2	1.04	TINF2	0.76	ADM	0.28
FASLG	1.04	STMN1	0.76	DDIT3	0.15
HMOX1	1.03	PPP1R15A	0.75		
PFKL	1.01	LIG4	0.74		

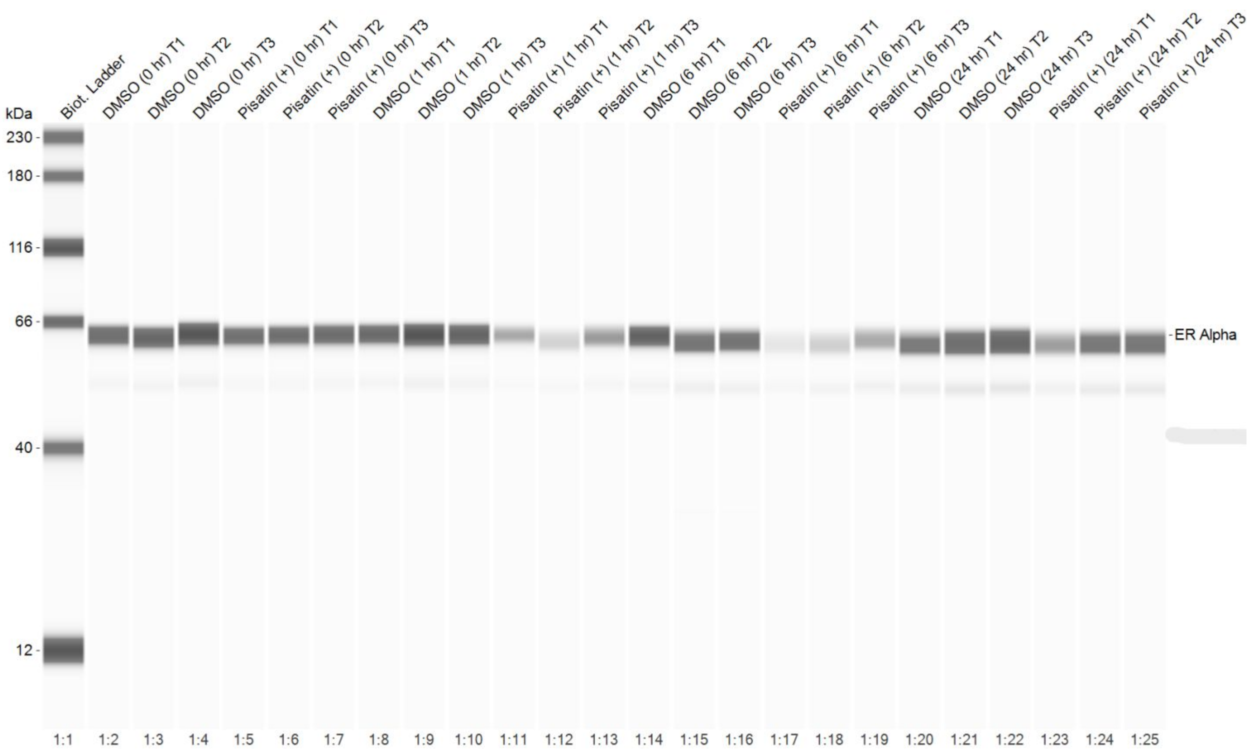

Figure S5: ERα JESS blot.

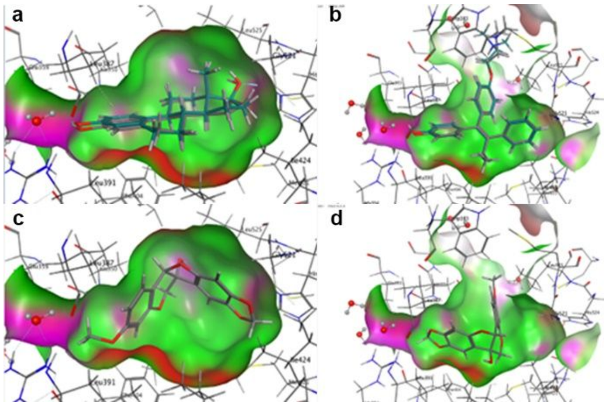

Figure S6. (+)-pisatin docking into the ERα pocket and the effect on estrogenic activity. (**a**) Estradiol (atom color) docked into 1ERE (left) and 4OH-tamoxifen (atom color) docked into 3ERT (right) superimposed on the corresponding ligand found in the crystal structure (blue); (**b**) (+)-pisatin docked into 1ERE (estradiol, agonist) pocket of ERα; **c**) (+)-pisatin docked into 3ERT (4OH-tamoxifen, antagonist) pocket of ERα; and **d**) the agonist pocket of the ERα pocket.

Table S3. Relative binding affinity of (+)-pisatin to ERα receptor.

	ER-α	
Compound	IC_{50}	RBA %
17β-estradiol	$7.00x10^{-10}$	100
(+)-pisatin	$2.15x10^{-7}$	0.326

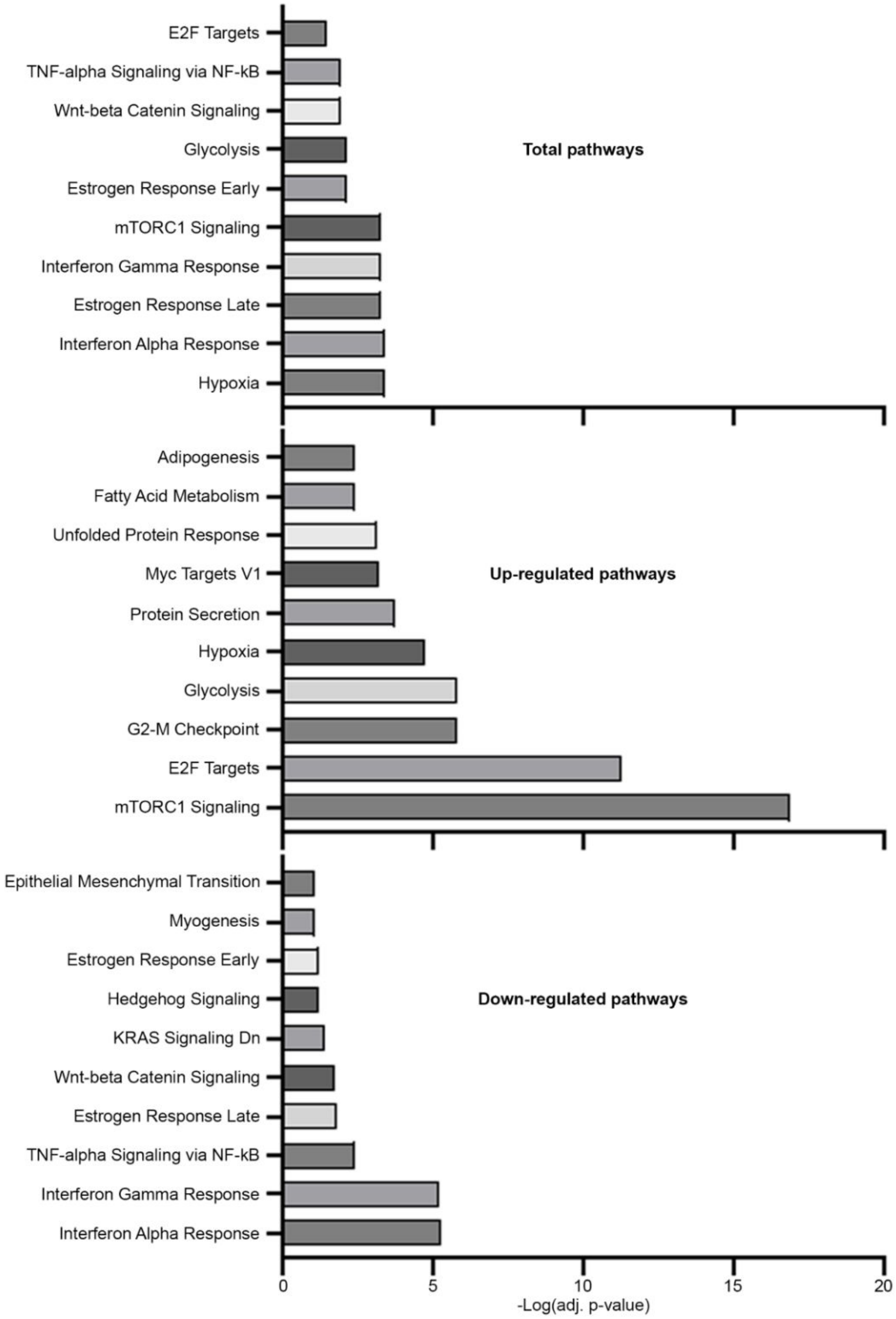

Figure S7: Comprehensive pathway analysis changes.

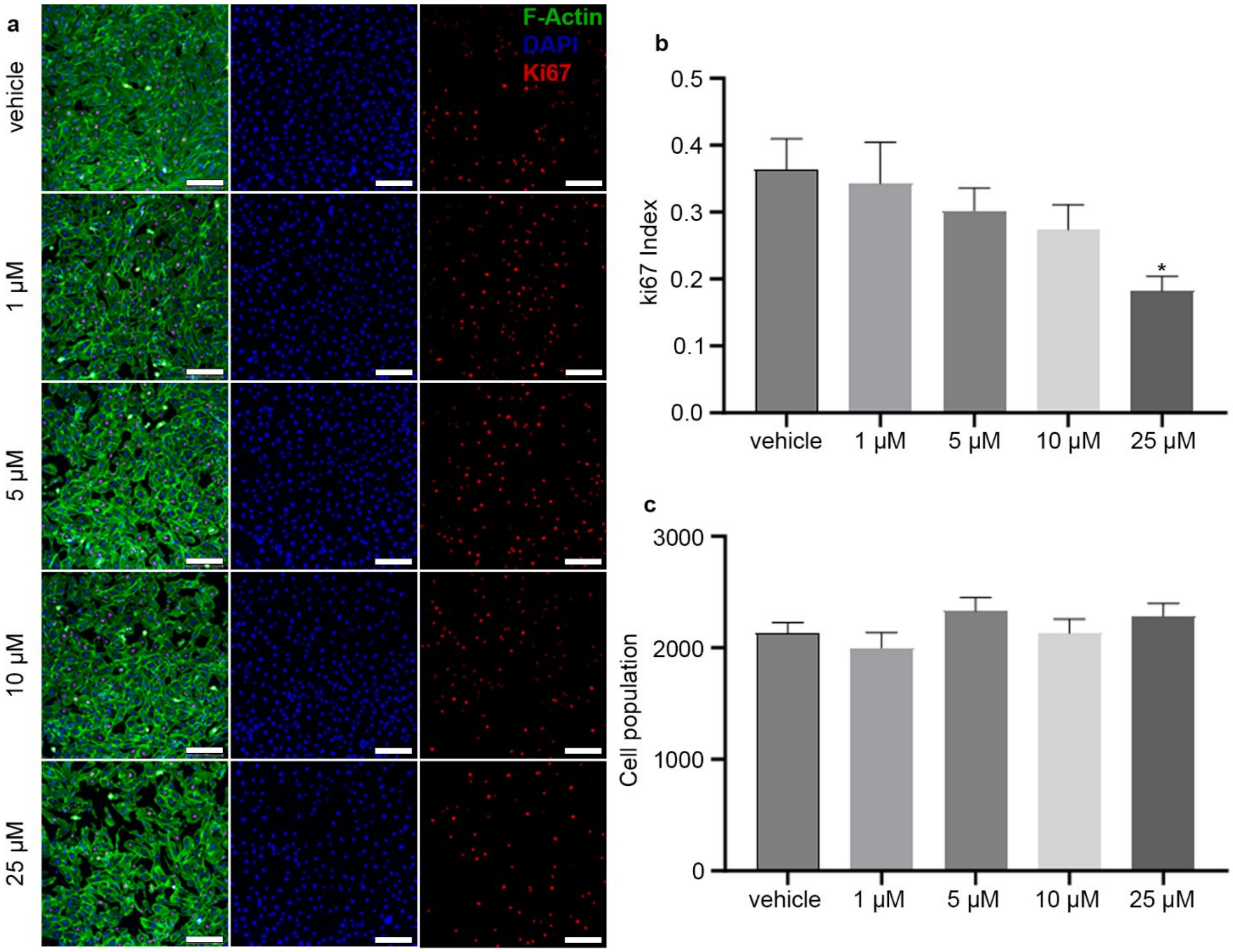

Figure S8. Dose-response of Human Umbilical Vein Endothelial cells after treatment with (+)-pisatin. a: Representative images of Laser Scanning Confocal Microscopy images of 2D-plated HUVECs treated with (+)-pisatin at 0uM, 1uM, 5uM, 10uM and 25uM concentrations for 24 hours. F-Actin in endothelial cells is labelled with phalloidins (green). Nuclei of all cells are labelled DAPI (blue). Ki67 is labelled Alexafluor 594 (red). Scale bar = 250um. b-c: ki67 proliferation index (b) and cell population (c) at 0uM, 1uM, 5uM, 10uM and 25uM (n=3). Statistical significance was analyzed using a One-way ANOVA with Dunnett's multiple comparison on GraphPad Prism (ns = non-significant, *p<0.05, **p<0.01, ***p<0.001, ****p<0.0001). Error bars represent SEM.
